# Engineered protein G variants for multifunctional antibody‐based assemblies

**DOI:** 10.1002/pro.70019

**Published:** 2025-01-25

**Authors:** Tomasz Slezak, Kelly M. O'Leary, Jinyang Li, Ahmed Rohaim, Elena K. Davydova, Anthony A. Kossiakoff

**Affiliations:** ^1^ Department of Biochemistry and Molecular Biology The University of Chicago Chicago Illinois USA; ^2^ Institute for Biophysical Dynamics The University of Chicago Chicago Illinois USA

**Keywords:** antibody engineering, bispecific antibodies, cell biology applications, engineered protein G, modular protein assembly

## Abstract

We have developed a portfolio of antibody‐based modules that can be prefabricated as standalone units and snapped together in plug‐and‐play fashion to create uniquely powerful multifunctional assemblies. The basic building blocks are derived from multiple pairs of native and modified Fab scaffolds and protein G (PG) variants engineered by phage display to introduce high pair‐wise specificity. The variety of possible Fab‐PG pairings provides a highly orthogonal system that can be exploited to perform challenging cell biology operations in a straightforward manner. The simplest manifestation allows multiplexed antigen detection using PG variants fused to fluorescently labeled SNAP‐tags. Moreover, Fabs can be readily attached to a PG‐Fc dimer module which acts as the core unit to produce plug‐and‐play IgG‐like assemblies, and the utility can be further expanded to produce bispecific analogs using the “knobs into holes” strategy. These core PG‐Fc dimer modules can be made and stored in bulk to produce off‐the‐shelf customized IgG entities in minutes, not days or weeks by just adding a Fab with the desired antigen specificity. In another application, the bispecific modalities form the building block for fabricating potent bispecific T‐cell engagers (BiTEs), demonstrating their efficacy in cancer cell‐killing assays. Additionally, the system can be adapted to include commercial antibodies as building blocks, greatly increasing the target space. Crystal structure analysis reveals that a few strategically positioned interactions engender the specificity between the Fab‐PG variant pairs, requiring minimal changes to match the scaffolds for different possible combinations. This plug‐and‐play platform offers a user‐friendly and versatile approach to enhance the functionality of antibody‐based reagents in cell biology research.

## 1 INTRODUCTION

Antibody‐based reagents play preeminently in numerous cell biology applications, including molecular detection in solutions or tissues, protein purification, ELISA assays, Western blotting, and therapeutic interventions. While these methods exhibit considerable variation in their procedures and functional readouts, they all share the fundamental reliance on antibodies as the core element. Researchers frequently depend on commercially available antibodies and design their experiments based on the accessibility of these reagents, which often can vary significantly in their quality (Couchman 2009). In many applications, antibodies need to be labeled in some form, either directly or by secondary antibodies, but characterizing their quality is often a frustrating and labor‐intensive process (Schumacher and Seitz 2016). Additionally, with the transformative advancement of powerful new microscopy and proteomics platforms, the importance of high‐performance antibody reagents in cell biology research has increased concomitantly.

Traditionally, immunization technologies for antibody generation (Köhler and Milstein 1975) have been supplemented by molecular display technologies with phage and yeast display methods (Boder and Wittrup 1997; Bradbury et al. 2011; Smith 1985; Viti et al. 2000). These recombinant approaches have broadened the utility of antibody‐based reagents significantly. Nevertheless, significant challenges still remain to fully utilize the potential of antibody reagents because many of the most prevalent applications involve functionalizing antibodies with cargos, like tags and reporter groups, through processes that are often costly, time‐consuming, and inefficient.

The most commonly employed methodologies for cargo attachment encompass chemical modifications to specific residue types or posttranslational modifications (Agarwal and Bertozzi 2015). However, these methodologies are inherently low throughput and can necessitate multiple purification steps to eliminate excess labeling agents and diminish the final product yield. These issues are further exacerbated by the fact that subsequent validation often reveals they have impaired functionality compared to their unmodified counterparts. With this backdrop, it was evident that there is a need to develop a new class of multifunctional antibody‐based affinity reagents with enhanced functionality and, importantly, be highly user‐friendly in their application.

To address these issues, a new technology platform using engineered Fab‐protein G (PG) modules has been developed. These modules can be combined in various formats to perform complex tasks beyond the capabilities of traditional antibodies. Protein G is a robust molecule that can be linked to a wide variety of cargos, from small detector molecules to large proteins. In our previous work, we used phage display mutagenesis to generate a PG variant (GA1) that had an ultra‐high affinity to a set of modified Fab scaffolds with no cross‐reactivity to an IgG Fc domain (Bailey et al. 2014). This provided the molecular framework whereby Fabs can bind to multiple GA1 domains that are linked together to form multivalent entities. Thus, multifunctional assemblies can be built akin to Lego blocks, pieces of which can be prefabricated as standalone units and then linked together in a plug‐and‐play fashion.

Herein, we describe a portfolio of synthetic protein G variants exhibiting selective binding characteristics that facilitate cargo attachment to multiple Fab scaffold types. The platform has been enhanced with the development of additional engineered Fab‐PG pairs, introducing orthogonality and expanding versatility across applications. PG variants are generated to recognize Fab scaffolds from different wildtype frameworks, enabling native Fabs from commercial sources to integrate into the system. The system allows for the attachment of cargo to multiple Fab scaffold types. For example, PG variants can be fused with fluorescently labeled SNAP‐tags and mixed with Fabs to detect multiple antigens in a single experiment. Fabs can also be attached to a module containing PG linked to a dimer Fc domain, producing an IgG‐like assembly. Furthermore, the system can be used to create bispecific antibodies and potent Bispecific T‐cell Engagers (BiTEs). We have also engineered PG variants to bind to the Fc domain, allowing cargos to be coupled in a similar fashion to the Fc domain of IgGs. Crystal structure analysis has revealed that the specificity between the Fab‐PG variant pairs requires few mutations, making it accessible to researchers with minimal molecular biology expertise. The PG constructs are easy to produce and can be stockpiled frozen or lyophilized as off‐the‐shelf reagents. Overall, this plug‐and‐play protein G‐based antibody platform simplifies many cell biology applications and increases throughput significantly.

## 2 RESULTS

### 2.1 Development of new protein‐G variants with Fab scaffold‐based selectivity

In previous work, an ultra‐high affinity (*K*_*D*_ of 100 pM) Fab scaffold (Fab^LRT^) was developed that binds the GA1 variant of protein‐G (PG) (Slezak et al. 2020). However, GA1 was originally generated for a mutated version of the Herceptin Fab scaffold (Fab^S^), it did not bind to wild‐type versions of the scaffold or any murine orthologs. More universal versions of GA1 could have significant value by allowing the plug‐and‐play capability of the system to be extended to facilitate applications exploiting multiplexing between other Fab scaffold types. Two categories of PG variants were envisioned. One would be fully universal being able to bind all native scaffolds broadly. The second category would bind with high affinity to a wild‐type (*wt*) human Fab (Fab^H^), but not to Fab^LRT^. This requirement would provide an orthogonal pair that could be used simultaneously in experiments that required the delivery of two different cargos.

#### 2.1.1 Generation of customized protein‐G binders to different Fab scaffolds

**FIGURE 1:**
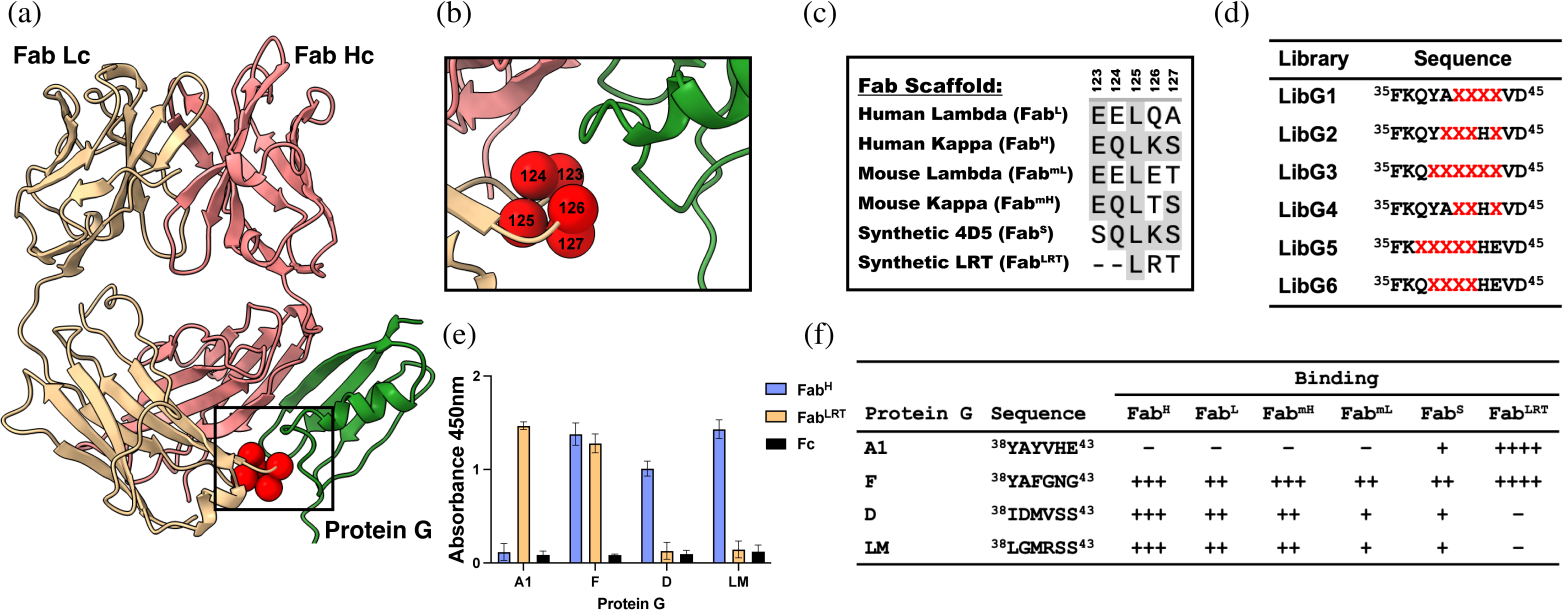
New protein G engineering. (a) The interface between the constant region of the Fab Light chain (Lc) and protein G (PG). Main interaction comes from an antiparallel β‐strand configuration of Fab Hc and PG. Additionally, PG interacts with Fab Lc via a α‐helical cap. Spheres represent randomized residues in the constant part of Fab Lc. Fab Hc is colored in red, while Lc is colored yellow. (b) The interaction between Fab Lc and PG is limited to 5 amino acids. Naturally existing Fab scaffolds contain conserved glutamic acid at position 123 and leucine at position 125. Interestingly, GA1 was previously engineered against Fab^S^, and does not recognize any of the naturally existing Fab scaffolds due to a negative charge at position 123. (c) Sequence alignment of constant part of the Lc (position 123–127) in different Fab scaffolds. The sequence alignment of Fab area recognized by PG shows the opportunity for affinity improvement. (d) Generated phage libraries where PG helical cap interacting with Fab Lc is randomized. Hard randomization (NNK) of selected residues is represented by “X.” (e) ELISA of selected PG variants against Fab^H^, Fab^LRT^, and Fc. Results show the high specificity towards certain Fab scaffolds that allow formation of orthogonal pairs that would not cross‐react (e.g., GA1‐Fab^LRT^ and GD‐Fab^H^). Protein GF is a universal high‐affinity Fab binder. Importantly, the interaction with the FC portion of the IgG has been engineered out. (f) The selected sequences of engineered PG variants. The binding affinities to different Fab scaffolds are indicated with “+” (++++: below 1 nM; +++: 1–10 nM; ++: 10–50 nM; +: more than 50 nM). No binding is indicated with “‐”. Sequences of all the generated PG variants are listed in Data S1.

To generate these scaffold‐specific PGs, we employed phage display mutagenesis using the GA1 variant as the starting point. Phage display libraries were designed focusing on the C‐terminal cap of the α‐helix in the heavy chain (Hc), central in the interaction with the constant portion of the Fab light chain (Lc) (Figure 1a,b). Figure 1c provides the sequences of the different Fab scaffold types at their point of engagement to GA1 (residues 123–127). Using Kunkel mutagenesis, we generated six libraries based on a hard randomized strategy (NNK diversity) covering residues 38–43 in the α‐helix region of GA1 (Figure 1d). Four of the libraries preserved His at position 42, since it had previously been found to improve the pH dependence of the engineered GA1 (Bailey et al. 2014). To enable possible conformational change in the helix, randomization of several residues bordering the helix that contacted the Fab Lc was introduced. The phage display selections involved using biotinylated Fabs as the target antigens.

##### Generation of the universal protein‐G variant

Five rounds of phage display selection were performed to generate a universal PG variant, sequentially swapping different Fab scaffolds from round to round. The 1st round targeted Fab^H^ (*wt* Herceptin scaffold having a kappa (κ) Lc), followed by Fab^L^ (human lambda (λ) Lc), Fab^LRT^, and Fab^S^ in successive rounds, ending with the final round repeating with the Fab^H^ antigen. Additionally, the antigen concentration was systematically reduced to increase stringency, starting with 1 μM in round 1 and ending with 1 nM in the final round. A phage ELISA was performed on 96 clones to validate the specificity of the selected PG variants. This resulted in the identification of seven universal PG variant candidates. Interestingly, a majority of the universal PG variants retained the *wt* PG composition at positions 42 and 43 (Figure S1, Supporting Information). This selection resulted in the universal GF variant (Figure 1e,f).

##### Generation of Fab^H^ PG variants

Next, we generated a set of PG variants recognizing wild‐type κ and λ Fab scaffolds (Fab^H^), but not the engineered ones (Fab^S^, Fab^LRT^). Fab^H^ is a wild‐type human (Herceptin) Fab with a κ Lc. The selection aimed to produce a PG variant that bound to human *wt* Fab scaffolds with both κ and λ Lc, but not the modified Fab^LRT^ scaffold. Five rounds of selection were performed using Fab^H^ as the target antigen. To counter‐select against binders to the Fab^LRT^ scaffold, 2 mM of unbiotinylated Fab^LRT^ was added to the selection buffer in each step of the biopanning. Thus, potential Fab^LRT^ binders were captured and eliminated in the wash steps, effectively depleting them from the library. The antigen concentration was systematically reduced round to round, starting with 1 mM and ending with 1 nM. After the final round of selection, phage ELISA was performed. The positive clones were sequenced, resulting in five unique high affinity Fab^H^ binders with no measurable cross reactivity to Fab^LRT^. Sequence alignment of these variants shows diversity was introduced in each position of PG with a dominating methionine at position 40 (Figure S1). Notably, although selections were made with a Fab having a κ Lc, both the GD and GLM variants showed varying cross‐reactivity towards human and murine Fabs with λ Lc (Figure 1e).

##### Biophysical characterization of the variant PGs

Surface plasmon resonance (SPR) was performed to determine the binding affinity and kinetics of the variant PGs. SPR showed a significant improvement in affinity and specificity compared to *wt* PG (Figures S2 and S3). The generation of multiple universal PGs was confirmed by testing the binding with the most common types of Fab scaffolds: Human κ (Fab^H^), Human λ (Fab^L^), Murine κ (Fab^mH^), and Murine λ (Fab^mL^), respectively (Tables 1, S2, and S3). The most promising universal PG candidate, GF, exhibits high affinity towards all tested Fab scaffolds—Fab^H^ (*K*_*D*_ 1.9 nM), Fab^L^ (*K*_*D*_ 21 nM), Fab^mH^ (*K*_*D*_ 3.2 nM), Fab^mL^ (*K*_*D*_ 37 nM), and Fab^LRT^ (*K*_*D*_ 0.9 nM). Additionally, the results indicate the successful generation of the orthogonal set of PG‐Fabs that would allow for simultaneous usage with the Fab^LRT^‐GA1 platform. Several generated PG variants are characterized by a high affinity and specificity towards Fab^H^ (Figure 1e) with the best candidates (GD, GLM) having affinities of 6.4 and 8.9 nM, respectively (Tables 1 and S2). The high specificity of the system was evaluated by ELISA, injecting 100 nM of Fab^LRT^ over the GD and GLM, confirming no detectable binding in either case (Figure 1e). Both GD and GLM recognized Fab^L^. However, the affinity was ~3‐fold lower than Fab^H^, which is consistent with the decreased affinity observed for GF and Fab^L^ (Table 1). A compilation of the specificity of PG variants for each scaffold type is presented in Figure 1f.

**TABLE 1:** Binding affinities of engineered protein G to different Fab scaffolds measured by SPR.

Protein G	Fab	*k*_on_ (M^−1^ s^−1^)	*k*_off_ (s^−1^)	*K*_D_ (nM)	*χ* ^2^ (RU^2^)
D	Fab^H^	8.3 × 10^5^	5.3 × 10^−3^	6.4	1.3
	Fab^L^	1.3 × 10^5^	2.1 × 10^3^	16	0.7
LM	Fab^H^	2.6 × 10^5^	2.2 × 10^3^	8.9	0.7
	Fab^L^	1.4 × 10^5^	3.0 × 10^−3^	21	2.1
F	Fab^H^	4.3 × 10^5^	8.2 × 10^−4^	1.9	0.2
	Fab^mH^	4.4 × 10^5^	1.4 × 10^−3^	3.2	0.4
	Fab^LRT^	5.2 × 10^5^	4.9 × 10^−4^	0.9	0.3
	Fab^L^	1.5 × 10^5^	3.2 × 10^3^	21	1.0
	Fab^mL^	1.3 × 10^5^	4.8 × 10^−3^	37	1.7

*Note*: *χ* ^2^ values are provided for all SPR runs. *χ* ^2^ is a measure of the averaged deviation of the experimental data from the fitting curve and it is accepted as a data quality indicator. All SPR sensograms are shown in Data S1. All SPR experiments were performed at room temperature.

Abbreviation: RU, response units.

#### 2.1.2 Structural insight into specificity differences between the protein GF and GD

The crystal structures of an antigen protein complexed with GF‐Fab^H^ and GD‐Fab^H^ described below were undertaken to establish the structural elements conferring the PG variants' individual specificities. The GF variant is “universal” in binding to all Fab formats. This contrasts with the GD variant, which does not bind to the engineered Fab^LRT^ scaffold, but all the natural human and murine scaffolds. In both cases, the target antigen protein used in the structure determination was the histone chaperone Anti‐silencing factor 1 (ASF1) protein (Bailey et al. 2018). The full complex contained the common components ASF1 and anti‐ASF1 Fab^H^ combined with either GF or GD to form a tri‐component complex. The anti‐ASF1 fab used here was fab E12 from the previous structure study (Bailey et al. 2018), but with its framework modified from Fab^S^ to Fab^H^. It is designated Fab E12^H^ for this set of structure determinations. The experimental particulars are provided in Table S1.

##### ASF1:Fab E12^H^ :GF

**FIGURE 2:**
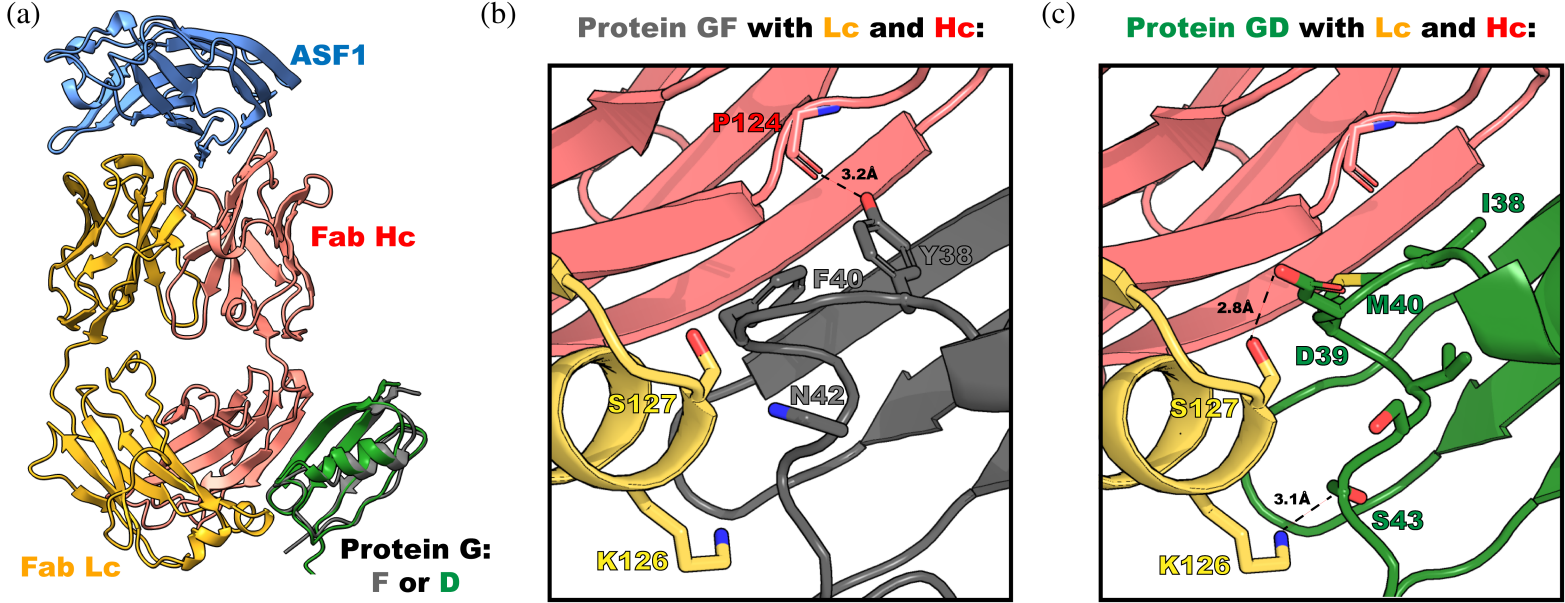
The structure of the ASF1‐E12 Fab^H^ with protein GF and protein GD. (a) General view of the structure. (b) Interface between protein GF and Fab^H^. Universal binding of GF comes from the extensive hydrophobic interactions of Y38, G41, and F40 with the Fab Hc. Additionally, Y38 forms a hydrogen bond with a main carbonyl of P124 from the Fab Hc. N42 placed itself between K126 and S127, forming the only interaction of GF with the Fab Lc. (c) Interface between protein GD and Fab^H^. The specificity of GD is driven by the set of H‐bonds formed by D39 and S43, which is disturbed by the significant loop rearrangement caused by the two amino acid deletion in Fab^LRT^. Side chains of S127 from Fab^H^ form hydrogen bonds with D39 from GD. Additionally, K126 forms hydrogen bond with a main chain carbonyl of S43 from GD. I38, D39 and M40 are engaged in several hydrophobic interactions with Fab Hc. M40 is placed in the hydrophobic pocket formed by P124, S125, V126, and V212 from the Fab Hc. I38 and D39 interact with S125 and F127 from Fab Hc, respectively. Molecules are colored as follows: ASF1, blue; Fab Hc, red; Fab Lc, orange; protein GF, gray; protein GD, green. PDB: (9AVO, 9AWE).

PG interacts with the Fab scaffold through an antiparallel β‐strand interaction with the Hc and a set of helical cap interactions with the Lc (Figure 2a). Since our protein engineering strategy did not involve altering this β‐strand contact, the H‐bonding in the strand was identical to the previously analyzed GA1‐Fab^LRT^ structure (Slezak et al. 2020). The main area of difference was the PG's helical cap interaction with Fab Lc, since the helical cap was broadly randomized in the PG phage display libraries. The Fab E12^H^‐GF interface is formed through two contacts that bury ~530 Å^2^ through its interaction with Hc and ~198 Å^2^ with Lc. Based on the buried surface area, most of the GF interaction surface emanates from the contact with the Fab's Hc, which includes an H‐bond formed between Y38 from GF and P124 of the Fab (Figure 2b). A further set of interactions is formed between Y38, G41, and F40 from GF with S125, F127, and V212 from the Fab Hc (Figure 2b). Notably, these Hc residues have conserved sequences among all the Fab frameworks, explaining the universality of the GF interactions (Slezak et al. 2020).

##### ASF1:Fab E12^H^ :GD

The GD variant is more selective than GF because it does not bind Fab^LRT^. The Fab^H^‐GD interface is formed through two principal contacts that bury ~596 Å^2^ for interaction with Hc and ~185 Å^2^ with Lc (Figure 2c). The specificity of GD comes from the set of hydrogen bonds formed by D39, S43, and D45 from the GD with Fab^H^ Lc. Side chains of S127 from Fab^H^ form H‐bonds with D39 from GD. Additionally, K126 forms two H‐bonds with a side chain of D45 and a main chain carbonyl of S43 from GD (Figure 2c). These interactions are eliminated by the significant loop rearrangement caused by the two amino acid deletions in Fab^LRT^ (Figure S4b). Interactions of I38, D39, and M40 from GD are formed with Fab Hc. M40 of GD is buried at the Hc Fab interface formed by P124, S125, V126, and V212 (Figure 2c). Additional interactions between I38 and D39 of GD with S125 and F127 from Fab Hc are formed, respectively.

Taken together, this structural information provides insight for the basis of the PG variant specificities. In the case of variant GA1, the inability to bind to Fab^H^ or other wt‐Fab Hc frameworks can readily be attributed to the charge repulsion between E43 of GA1 and E123, which is present in all wt‐Fab Hc molecules (Figure S4a). The inability of Fab^LRT^ to bind to the GD variant can be directly attributed to the two amino acid deletion in the Fab^LRT^'s Lc, which induces a significant loop rearrangement which places its T127 side chain into steric conflict with D39 of the GD variant (Figure S4b).

#### 2.1.3 Applications exploiting the modular design of PG variants

The PG‐Fab plug‐and‐play system described below was developed to provide researchers with a highly diverse toolkit of antibody‐based reagents that can be readily applied across many different types of applications. This system overcomes many of the limitations that have plagued the general use of traditional antibodies in some complex applications. The basis of the plug‐and‐play system is that highly customized affinity reagents can be constructed from simple component parts that can be assembled like Lego blocks by taking advantage of the ultra‐high affinity PGs with their orthogonal Fab scaffolds. Thus, multifunctional assemblies that would be highly challenging to produce as single entities can be readily fabricated with interchangeable parts that easily alter specificity and avidity to match the particular application.

##### Stability of plug‐and‐play PG complexes in cell‐based applications

The modular design that allows for the ability to “snap‐on” functional cargo onto antibodies and antibody fragments in a facile manner has obvious applications and is advantageous over traditional chemical coupling methods and protein fusions. This modular design concept also provides easy multiplexing, allowing for high throughput processing of many samples with minimal system changes. One consideration, however, is that the attachment is non‐covalent and there might be a concern that the cargo could disengage during the experiment's lifetime. To better quantify the degree of dissociation under typical experimental conditions, we undertook a time‐course analysis of the stability of a typical PG variant‐Fab complex. This involved measuring the changes in binding on HeLa cells of an EGFR Fab with a human kappa scaffold (Fab^H^) premixed in a 1:1 molar ratio with the PG variant, GLM, that was labeled with Alexa647. The cells were rigorously washed and the binding of the EGFR Fab was visualized using Flow Cytometry over a time interval of 24 h. The cells were kept at 4°C during the experiment to minimize receptor internalization. Figure S5 shows the average mean fluorescence intensity (MFI) observed over six time points from 0 min to 24 h is virtually unchanged over the full‐time course. For this analysis, we selected a Fab‐PG variant combination (Fab^H^‐GLM) that was one of the lower affinity pairs (9 nM). Most of our cell biology applications utilize the Fab^LRT^‐GA1 pairing, which has an affinity about 100‐fold higher (~0.1 nM). Thus, even using the lower affinity pair, the plug‐and‐play module is as functionally effective as the covalent alternatives during the time course of most typical cell biology experiments.

##### Generation of the Fc specific protein‐G variant

**FIGURE 3:**
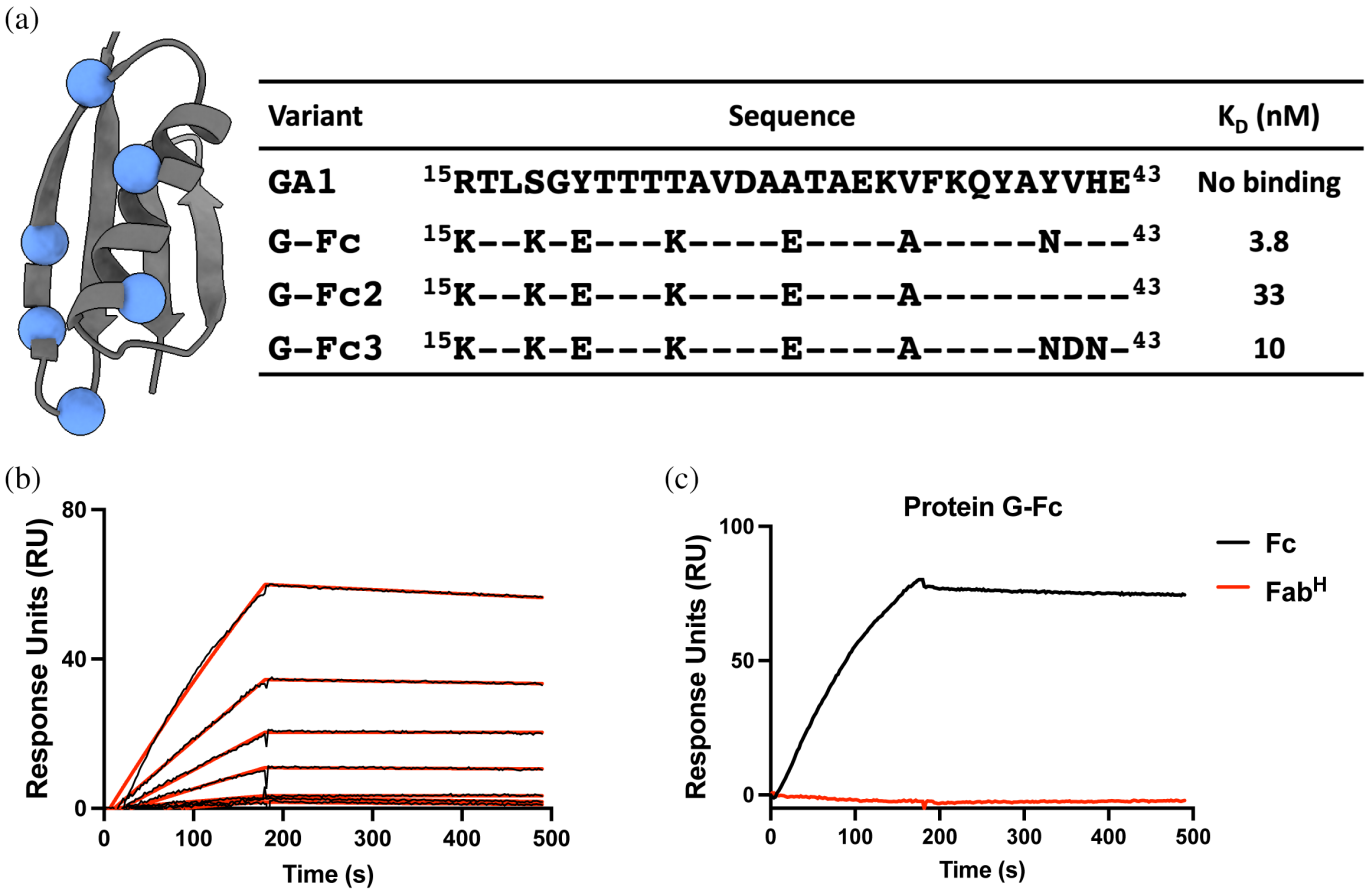
Engineering of protein G‐Fc. (a) The sequences of protein G‐Fc variants. Residues mutated to generate a protein G‐Fc are represented as blue spheres. (b) SPR sensogram showing the interaction of G‐Fc with human Fc. For the kinetic experiment, analytes were serially diluted two‐fold, starting at 100 nM. (c) A single injection of Fc and Fab^H^ on G‐Fc. 200 nM of Fc and 200 nM of Fab^H^ were injected, and no binding to Fab^H^ was observed. SPR experiments were performed at room temperature.

Many cell‐based experiments utilize commercially available antibodies in IgG format to exploit avidity effects, cell killing properties and detection by off‐the‐shelf secondary antibodies. Thus, we endeavored to develop a PG scaffold that could be used in a plug‐and‐play fashion with any full‐length antibody. This allows myriad cargo types to be facilely attached to any commercial human IgG. *Wt* protein G binds to IgG molecules through both the antibody's Fab and Fc domains. For most applications, it is important to separate the binding between the Fab and Fc domains to eliminate undesired cross‐reactivity that might interfere with interpretations in many cell based experiments. The PG variants described above were engineered to eliminate any binding to an IgG Fc domain. To accomplish this, the original GA1 variant from which the specificity matured GF, GD, and GLM variants were derived, included a set of mutations introduced to specifically inhibit binding to the Fc portion of an IgG antibody. To convert GA1 into an Fc specific binder, a reverse engineering strategy was used. Based on the previously published structure of the *wt* protein G in complex with the Fc domain of human IgG (Sauer‐Eriksson et al. 1995), we selected seven residues in GA1 that had originally been mutated to eliminate Fc binding and reverted their sequences back to *wt* PG (Figure 3a). Three separate variants were created by making further mutations in the helix cap region of PG. Binding kinetics were determined by SPR, showing the successful generation of G‐Fc, which possesses a low nM affinity to the Fc domain (3.8 nM) with a prolonged dissociation rate (Figure 3b and Table S4). A 200 nM injection of Fab^H^ tested the high specificity of the G‐Fc by showing no detectable binding to the Fab was observed (Figure 3c). Variants G‐Fc2 and G‐Fc3 displayed somewhat lower binding affinities and less optimal kinetics; thus, all our experiments were performed with the G‐Fc variant.

##### Plug‐and‐play IgG

Due to their avidity effect, enhanced binding affinity is one advantage of bivalent IgGs over monovalent Fabs. In this regard, fusing together multiple GA1 domains like beads on a string can be exploited to increase the binding of cargo to immobilized targets, as shown in Figure S6, where both the affinity and kinetics are substantially improved going from mono‐ to bi‐ to tri‐valent modalities: 15‐fold, 50‐fold, respectively. The constructs are very versatile in that the number of domains and linker lengths between them are easily modified and thus, can be customized to the particular application. While the ability to use linked GA1 domains systematically increases the affinity of the construct to its designated target, it lacks the functional attributes innate in Fc domains of natural antibodies. Thus, we thought that a significant enhancement would be to fabricate a plug‐and‐play IgG‐like molecule (G_Fc_). Conceptually, this could facilitate the facile interchange of Fab components through the PG linking technology while having the advantages of the structure and function of natural antibody molecules.

**FIGURE 4:**
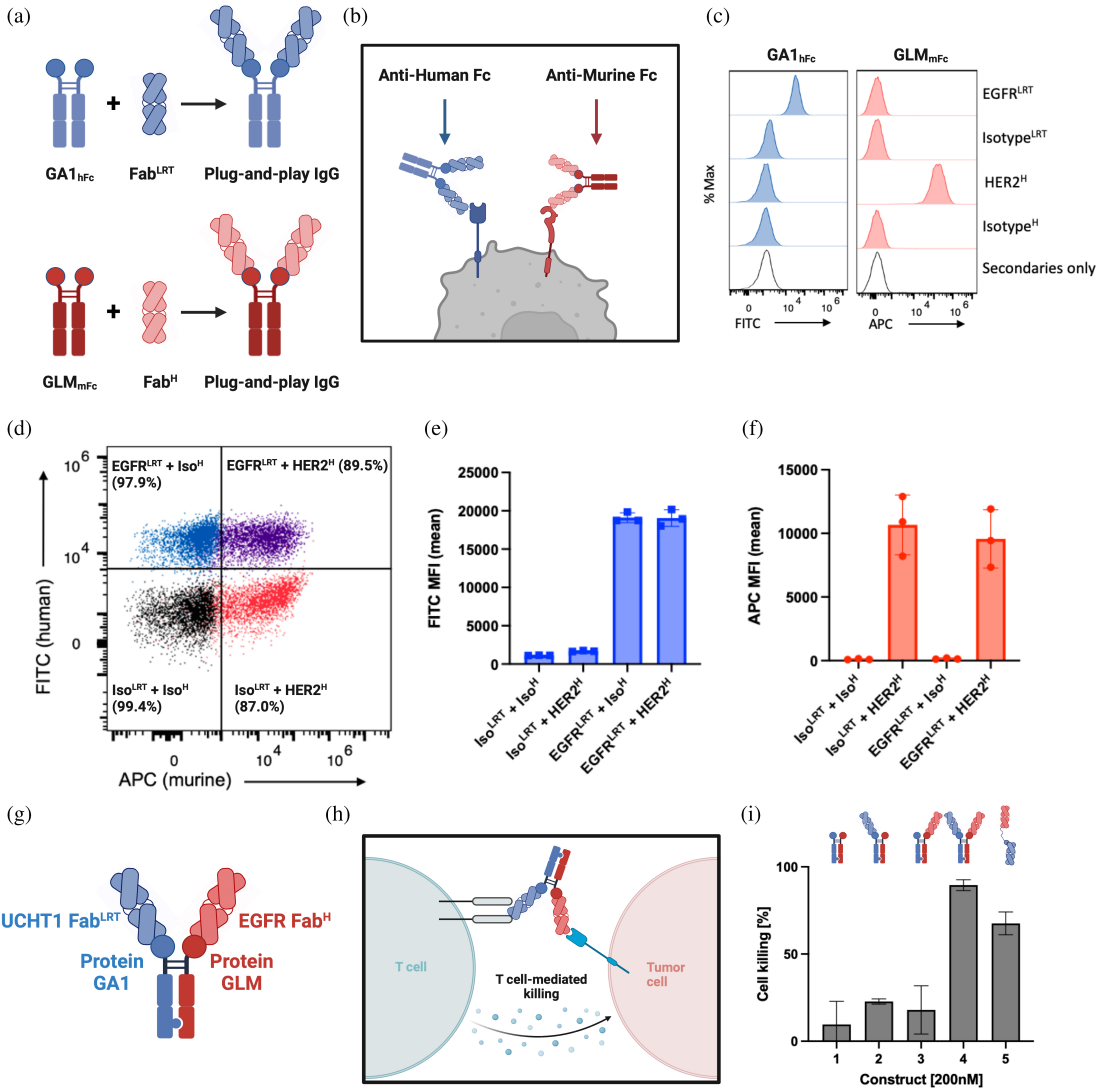
Protein G_Fc_ fusion enables modular assembly of bivalent IgG‐like molecules. (a) Schematic of plug‐and‐play IgG assembly with human or murine Fc using orthogonal PG‐Fab scaffold pairs. (b) Orthogonal PG_Fc_ fusions enable simultaneous detection of two different sABs binding to the cell surface. Model for secondary detection by anti‐Fc secondary antibodies that recognize either human IgG1 Fc or murine IgG2a Fc. (c) HCC1954 cells were stained with IgG‐like sABs targeting EGFR (Fab^LRT^ format) or HER2 (Fab^H^ format). Left panel, anti‐human‐Fc‐A488 (FITC) recognizes only the combination of GA1_hFc_ + EGFR^LRT^. Right panel, anti‐murine‐Fc‐A647 (APC) recognizes only the combination of GLM_mFc_ + HER2^H^. (d) Simultaneous staining of EGFR and HER2 using the IgG‐like assemblies shown in (C). (e) Quantification of mean fluorescence index (MFI) in the FITC channel to visualize EGFR^LRT^ binding. (f) Quantification of MFI in the APC channel to visualize HER2^H^ binding. (g) Plug‐and‐play bispecific antibodies (bi‐IgG). Schematic of plug‐and‐play bispecific IgG‐like assembly. “Knobs‐into‐holes” heterodimerization technology allows for the generation of Fc‐fusion dual PG molecule which contains engineered protein GA1 and GLM that allow for the specific recognition of Fab^LRT^ or Fab^H^, respectively. (h) Model of cell killing experiment. One Fab recognizes EGFR extracellular domain on the antigen presenting cells (APC), while the second Fab binds to CD3 of the T‐cell receptor. (i) The effect of bi‐IgG molecule on PBMC/PC3 (10:1) co‐cultures. Constructs were added at 200 nM concentration and the effect of cell killing was measured using LDH release assay after 24 h. As a control, all the individual components of the system (lane 1, 2, and 3) were tested and showed a marginal cell killing, while the robust effect was observed when both components of the system were present (lane 4). As a positive control, previously published plug‐and‐play BiTE technology was used (lane 5). Components of lanes: 1. GA1_Fc_GLM not loaded with Fabs; 2. GA1_Fc_GLM + UCHT1 Fab^LRT^; 3. GA1_Fc_GLM + EGFR Fab^H^; 4. GA1_Fc_GLM + UCHT1 Fab^LRT^ + EGFR Fab^H^; 5. plug‐and‐play BiTE (OKT3‐GA1) + EGFR Fab^LRT^.

The basic PG‐IgG framework fabrication is straightforward and retains all elements of a natural antibody. The construction involves fusing two PG arms to an IgG Fc base; the PG arms can be any of the variants described above to provide a panel of different specificities. Fabs having a particular PG specificity can be simply introduced to mimic the full‐length bivalent IgG (Figure 4a). The linker lengths between the PG domains and the Fc fragment can be adjusted to optimize the molecule's efficacy with respect to a particular application. The initial test of this assembly was performed using two GA1s fused to a human Fc domain via a 17 residue linker (GA1_Fc_). This linker length is similar to that found in natural antibodies. A conformation‐specific anti‐maltose binding protein (MBP) Fab^LRT^ (7O Fab^LRT^) was associated with the Fc through its GA1 arm to detect extracellular MBP that had been stably expressed on the surface of the HEK cell line (Figure S7). An attribute of using the conformational specific 7O Fab was that it also provided an adjustable binding switch that could be controlled by systematically altering the concentration of maltose. Using an ELISA assay, we determined that 7O in its Fab format in the absence of maltose bound to cells with an EC_50_ value of 10 nM compared to ~3 nM in the GA1_Fc_ bi‐valent format, demonstrating the bivalent avidity effect. In the presence of 10 μM maltose, no binding was observed for the monovalent Fab compared to 85 nM for the GA1_Fc_ format (Figure S8).

Next, we asked if we could generate a set of plug‐and‐play IgGs to facilitate the orthogonal pairs of GA1‐Fab^LRT^ and GLM‐Fab^H^, allowing for the co‐binding detection of two antibodies (Figure 4b). To do so, we linked GLM to a murine Fc fragment and GA1 to a human one, creating the set of reagents that could be detected using anti‐Human Fc or anti‐Murine Fc secondary antibody. We performed a similar co‐binding experiment using the HCC1954 cell line and two model cell surface receptors, HER2 and EGFR. Anti‐Her2 Fab was grafted into Fab^H^, while Fab^LRT^ scaffold was introduced into anti‐EGFR Fab prior to mixing with GLM_Fc_ or GA1_Fc_, respectively. The binding of anti‐Her2 Fab^H^ + GLM_Fc_ and anti‐EGFR Fab^LRT^ + GA1_Fc_ were detected using anti‐murine‐Fc‐A647 or anti‐human‐Fc‐A488, respectively. This resulted in the efficient detection of the simultaneous binding of EGFR and HER2 plug‐and‐play IgGs, which was recorded when co‐staining was tested (Figure 4c). The controls of Isotype Fabs in each configuration did not produce detectable binding, proving the system's efficiency (Figure 4d). We did not detect any exchange between the components of the system (Figures 4e,f and S10).

##### Plug‐and‐play bispecific antibodies (bi‐IgG)

Further, using “knobs‐into‐holes” heterodimerization technology (Von Kreudenstein et al. 2013), we generated a bispecific IgG‐like assembly that provides for the orthogonal usage of Fab^LRT^ and Fab^H^ (Figure 4g). The body of the Fc unit comprises an Hc component containing a GLM arm that binds specifically to Fab^H^ and a second Hc component composed of a GA1 arm that binds Fab^LRT^. The specificities of the arms prevent any possible interchanging the Fabs.

To test the efficiency of this bi‐IgG assembly, we engineered a bispecific T‐cell engager (BiTE) using the combination of the GLM and GA1 Hc components (Figure 4h). The bi‐IgG forces the engagement of an antigen presenting cancer cell (APC) with a cytotoxic T‐cell. The APC in this experiment was a PC3 cancer cell highly expressing EGFR on its cell surface; this cell was targeted by an anti‐EGFR Fab^H^ binding through the GLM arm. The second arm consisted of the GA1 variant, which binds to an anti‐CD3 Fab^LRT^ (UCHT1) (Figure 4g). This targets and activates the T‐cell receptor on a T‐cell. The efficiency of the bi‐IgG BiTE was determined by measuring the activity of the cytoplasmic enzyme lactate dehydrogenase (LDH), which gets released into the medium upon cell killing. The addition of bi‐IgG that contained all the active components to PBMC‐PC3 co‐cultures at 200 nM, resulted in robust cell killing (Figure 4i). Importantly, no effect was observed when only one of the arms of bi‐IgG was present in the assembly (Figure 4i). Notably, the functional readout was similar to the positive control that applied OKT3 antibody in the previously described plug‐and‐play bi‐Fab format (Slezak et al. 2020).

##### Secondary reagents for antibody binding detection by microscopy and flow cytometry

Co‐staining of multiple targets using traditional antibodies for flow cytometry or microscopy experiments involves a careful selection of secondary detection reagents, often requiring the primary antibodies to be from different species to avoid cross‐reactivity. In this regard, the high specificity of the engineered PG variants and the ease with which fluorescent labels can be attached to them makes them good candidates for myriad cell biology applications. To evaluate their performance, it was first important to assess the detection sensitivity of the system. The model system we used was simple and controllable, consisting of a GA1‐SNAP tag fusion labeled with Alexa 647. It was then complexed to a Fab that recognizes the unliganded state of MBP, which had been engineered to be expressed on the surface of HEK293 cells. The anti‐MBP Fab (7O) was converted to the Fab^LRT^ framework, providing a 100 pM binding affinity to the GA1‐A647 modality. This high affinity between GA1‐Fab^LRT^ allows for simply premixing all reagents prior to the staining protocol, eliminating an additional washing step and significantly shortening the procedure time. This model system allows for additional experimental control since the conformational change of MBP upon maltose binding results in the modulating 7O binding in a controlled way. Thus, the detection of an Alexa 647 signal in a flow cytometry experiment can be evaluated in a concentration‐dependent manner and completely eliminated by the spike of 1 mM maltose (Figure S7).

**FIGURE 5:**
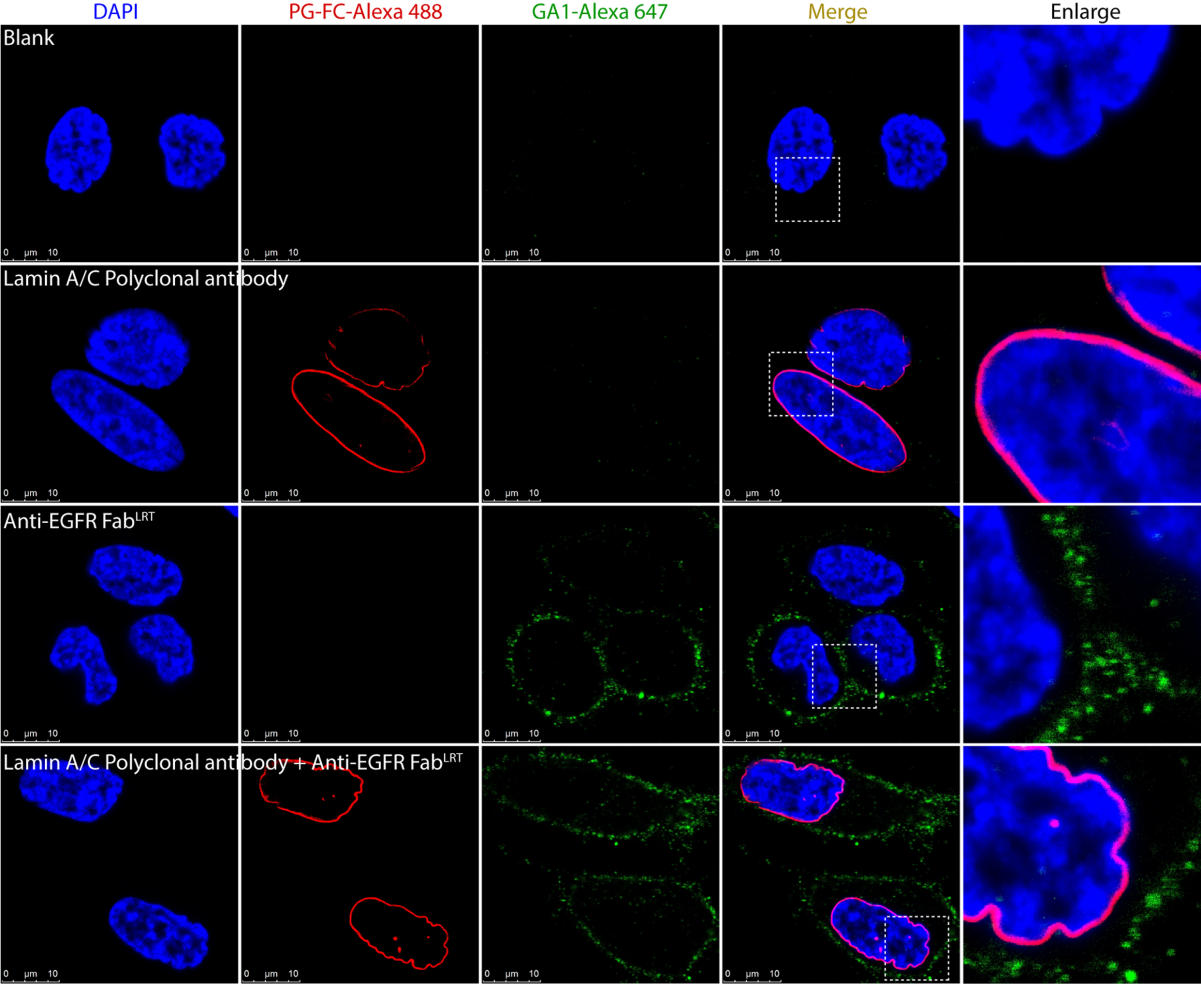
Engineered protein G variants enable simultaneous immunofluorescent co‐staining using synthetic Fab^LRT^ and commercially available IgG. Anti‐EGFR Fab^LRT^ was premixed with Alexa647‐labeled GA1 and anti‐Lamin A/C Rabbit IgG was premixed with Alexa488‐labeled protein G‐Fc prior to immunofluorescent HeLa cell staining. Confocal imaging revealed EGFR signal is equally distributed at the plasma membrane while Lamin A/C signal is concentrated around the nuclear envelope. The orthogonal interactions of Alexa647‐GA1 with anti‐EGFR Fab^LRT^ and Alexa488‐G‐Fc with anti‐Lamin A/C Rabbit IgG exhibited high specificity and allowed for simultaneous co‐detection of two distinct cellular antigens.

To further extend the utility of the approach, we next evaluated the feasibility of attaching SNAP G‐Fc to commercial antibodies. Since most of the community that routinely uses antibodies relies on commercially available IgGs, we posited that the SNAP G‐Fc modality would be broadly useful by providing an off‐the‐shelf means to label virtually any antibody derived from multiple species. We used a commercially available Lamin A/C Rabbit IgG for the test case. SNAP G‐Fc and SNAP GA1 were labeled with Alexa 488 and Alexa 647 via SNAP‐tag, respectively and combined with their respective antibody format. Hela cells were stained with anti‐EGFR Fab^LRT^ for 15 min on ice to prevent the internalization of EGFR. The cells were then washed, fixed and permeabilized, followed by the staining with anti‐Lamin A/C IgG. The binding of anti‐EGFR and anti‐Lamin A/C was detected with SNAP GA1‐A647 and SNAP G‐Fc‐A488. The signal for anti‐EGFR Fab^LRT^ was nicely distributed in the cell plasma membrane, while the Lamin A/C was concentrated around the nuclear envelope (Figure 5).

Similar results were obtained for the co‐binding detection of two fluorescently labeled Fabs that recognize different cell surface targets (Figure S9a). As a proof‐of‐concept, we chose to co‐stain the HER2 and EGFR on SKBR3 cells, exploiting the ability to selectively match Fab scaffold types with their cognate PG variant partners. Before cell staining, SNAP GA1‐A647 and SNAP GLM‐A488 were premixed with anti‐EGFR Fab^LRT^ and anti‐HER2 Fab^H^ to form the two secondary detection agents. The binding of anti‐EGFR and anti‐HER2 was detected by SNAP GA1‐A647 and SNAP GLM‐A488 both independently and simultaneously (Figure S9b,c). Conversely, no co‐staining of the cells was observed when the control isotype Fabs against Ebola nucleoprotein and MBP in analogous Fab‐PG formats were used (Figure S9b). Not surprisingly, the fusion of double SNAP tag to GA1 significantly improved signal to noise ratio of the system (Figures S9d and S11).

## 3 DISCUSSION

We have designed and developed a versatile toolkit of powerful antibody‐based reagents. These reagents can be assembled from independent functional component parts in a plug‐and‐play fashion using minimal molecular biology manipulation. These reagents, based on protein G (PG)‐Fab assemblies, function like Lego blocks, allowing for the creation of multi‐specific and multi‐functional targeting moieties. This modular approach enables diverse antibody‐cargo combinations. Previously, high‐affinity PG‐Fab modules were developed using phage display, leading to ultra‐high affinity modules for efficient cargo attachment (Slezak et al. 2020). The stability of these assemblies is directly dependent on the affinities between the component parts (Figure S13), which is sufficient for most in vitro cell‐based assays. The toolkit has been expanded to include complementary and orthogonal Fab‐PG pairs with distinct targeting properties, expanding application possibilities. New PG variants were created to bind specific wild‐type human and murine Fab scaffolds, enhancing versatility. The toolkit's orthogonal PG and Fab pairs can be used interchangeably, allowing for various combinations to be evaluated in a multiplexed fashion. Constructs can range from simple linear arrangements linking multiple copies of a PG variant, like beads on a string, to complex multi‐specific assemblies. Further, PG variants are robust molecules to which various types of cargo can be readily fused (Slezak et al. 2020).

In cell biology, precise antigen detection is crucial. PG‐Fab modules can lead to enhanced detection by attaching SNAP‐tags or fluorescent proteins to PG variants, improving signal‐to‐noise ratios compared to traditional antibodies and simplifying experimental design by reducing cross‐talk issues. The modular aspect of the PG variants allows for the fabrication IgG‐like constructs containing PG variants linked to Fc fragments, facilitating quick assembly from Fab to IgG formats. Bispecific PG‐Fab moieties can be created using conventional heterodimerization strategies, enabling applications like bispecific T cell engagers (BiTEs) that show potent cell‐killing capabilities. Additionally, the platform can exploit commercially available antibodies by reverse engineering the PG scaffold to restore Fc binding capabilities. This allows for efficient attachment of various cargos and the creation of super avidity assemblies by linking multiple IgGs.

The examples presented demonstrate the potential scope of experiments that can be enabled by the ability to snap together easy to manipulate component parts to build highly diverse antibody‐cargo combinations. This strategy offers a user‐friendly approach to constructing diverse antibody‐cargo combinations with minimal expertise required. The modular assemblies can be produced in bulk and stored as off‐the‐shelf reagents for future experiments or high‐throughput multiplexing across antibody cohorts.

## 4 MATERIALS AND METHODS

### 4.1 Protein cloning, expression, and purification

The sequences of all used constructs are provided in Table S5. Protein Gs cloning strategy was previously described (Slezak et al. 2020). Briefly, the protein Gs were cloned using Sma1 site into pEKD40 with the Thrombin cleavable N‐terminal SNAP‐tag and C‐terminal His‐tag. Fabs. All Fab^LRT^ scaffold was grafted into Fab Lc at aa positions 123–127 (SQLKS → LRT) using quick change site‐directed mutagenesis. Protein Gs were expressed in BL21 (DE3) grown overnight in 2xYT medium at 20°C post induction with 1 mM IPTG at OD_600_ = 0.6. Cells were sonicated in buffer A containing 50 mM Tris–HCl, pH 8.0, 150 mM NaCl and 10% glycerol. His‐tagged protein was purified from the supernatant post centrifugation using Talon (TaKaRa, Cat # 635653) cobalt resin and eluted with 100 mM imidazole in buffer A.

Fabs were expressed in the periplasm of *Escherichia coli* BL21 cells for 4 h at 37°C post induction with 1 mM IPTG at OD_600_ = 0.8–1. The cells were harvested by sonication in protein G‐wash buffer (Bailey et al. 2014) (50 mM Phosphate buffer, 500 mM NaCl, pH 7.4). After centrifugation, the supernatant was applied on the protein GF affinity column (Figure S12). Proteins were eluted from the column with 0.1 M glycine, pH 2.6, and neutralized with 1M Tris–HCl, pH 8.5.

Fc fusion proteins were cloned into the pSCSTa vector containing a human or murine IgG construct. First, the vector was linearized via PCR designed to remove the CH1 portion. GA1 was amplified by PCR to contain a C‐terminal linker (GGGGGGSGGGGSGGGGSSSGSS) and was then cloned into the N‐terminal portion of the CH2‐CH3 construct remaining in the open vector. GLM_hFc_ and GLM_mFc_ were generated by site‐directed mutagenesis using GA1‐hFc and GA1‐mFc as templates. PG_Fc_ fusions were produced by transfection of Expi293 cells (ExpiFectamine, Gibco, Cat # A14525) according to manufacturer's recommendation and were purified with protein A resin.

### 4.2 Protein GF resin preparation

Protein GF resin was generated as previously described (Bailey et al. 2014). Briefly, protein GF was cloned with a SUMO‐tag, that contains a free cysteine to allow for covalent linkage to SulfoLink Coupling Resin (Thermo Scientific, Cat # 20401), and the resin was created following the manufacturer's protocol.

### 4.3 Phage display library preparation

The library generation strategy was designed using a previously described approach (Bailey et al. 2014; Sidhu et al. 2000). Protein G was displayed on the surface of M13 phage by fusion to the minor coat protein pIII. After the inspection of the Fab‐protein G crystal structures (1IGC and 6U8C) the 6 amino acids at the position 38–43 were identified to interact with the Fab constant Lc. These residues were randomized using hard randomization strategy (NNK) where all amino acids are possible. Stop codon was placed using quick change site‐directed mutagenesis in the aa position 40 prior to ssDNA preparation from phage. Phosphorylated primers were used in Kunkel mutagenesis (Kunkel 1985) and the library was created as described before (Slezak and Kossiakoff 2021).

### 4.4 Phage display selection

Prior to the phage display biopanning, the target fab scaffolds with Avi‐tag were expressed, purified and biotinylated with BirA enzyme. In the first round of selection, 1 μM of Fab^H^ (Human Kappa) was immobilized on 200 μL SA magnetic beads (Promega, Cat # Z5482) and incubated with 1 mL phage library for 1 h at room temperature with gentle shaking. Each of the generated phage libraries was used separately. The beads were washed three times to remove nonspecific phage and added to log phase *E. coli* XL‐1 blue cells (Stratagene, Cat # 200249) and incubated for 20 min at room temperature. Then, media containing 100 μg/mL ampicillin and 10^9^ p.f.u./mL of M13K07 helper phage (NEB, Cat # N0315S) was added for overnight phage amplification at 37°C. For all subsequent rounds, the amplified phage was precipitated in 20% PEG/2.5M NaCl for 20 min on ice. Before each round, the phage pool was negatively selected against empty paramagnetic beads for 30 min with shaking to eliminate nonspecific binders. The final concentration of antigen was dropped gradually from 1 μM to 1 nM from the first to the fifth round (2nd round: 200 nM, 3rd round: 50 nM, 4th round 10 nM and 5th round 1 nM). After phage binding, the beads were subjected to five washing rounds. The bound phages were eluted using 0.1M glycine, pH 2.6 and neutralized with TRIS–HCl, pH 8.5. Then, the phage eluate was used for *E. coli* infection and phage amplification, as described above. After rounds 4 and 5 phage, infected bacteria were plated on ampicillin plates and 96 single colonies were picked for single‐point phage ELISA assays. The promising clones demonstrating high ELISA signal and low non‐specific binding were sequenced and reformatted into a pEKD40 expressing vector, at room temperature overnight, as described in protein cloning, expression and purification. To generate the universally binding protein G, the different Fab scaffolds were introduced as a target in consecutive rounds of selection (1st round: Fab^H^, 2nd round: Fab^L^ (Human Lambda), 3rd round: Fab^LRT^, 4th round: Fab^S^ (4D5), 5th round: Fab^H^). The selection procedure was then followed, as described above.

### 4.5 Enzyme‐linked immunoabsorbent assays

Enzyme‐linked immunoabsorbent assays (ELISA) protocol was described before by Lan et al. (2024). Briefly, 50 nM of different Fab scaffolds were directly immobilized on high binding experimental wells (Greiner Bio, Cat # 655061) and BSA was immobilized in control wells, followed by extensive blocking with BSA. After 15 min incubation with phage, wells were extensively washed three times and incubated with protein L‐HRP (Thermo Scientific, Cat # 10321504, 1:5000 dilution in HBST) for 20 min. The plates were again washed and developed with TMB substrate (Thermo Scientific, Cat # N301) and quenched with 10% H_3_PO_4_, followed by the absorbance at A_450_ determination.

### 4.6 Multipoint ELISA

High‐binding experimental wells (Greiner Bio, Cat # 655061) were used to immobilize MBP at a concentration of 50 nM. The wells were extensively blocked with BSA. Twelve 2‐fold serial dilutions were added and incubated for 20 min at room temperature for each construct being analyzed. The wells were then subjected to extensive washing before being incubated with HRP‐conjugated anti‐human (Fab)2 antibody (JacksonImmunoResearch, Cat # 109‐036‐003) at a dilution of 1:5000 in PBST for 20 min at room temperature. After washing again, the wells were developed with TMB substrate (Thermo Scientific, Cat # N301), and the reaction was quenched with 10% H3PO4. The absorbance at A450 was then determined.

### 4.7 Surface plasmon resonance analysis

All surface plasmon resonance (SPR) analysis were performed on a MASS‐1 (Bruker). All targets were immobilized via a 6x His‐tag to a Ni‐NTA sensor chip. Fabs in twofold dilutions were run as analytes at 30 μl/min flow rate at 20°C. Sensograms were corrected through double referencing and a 1:1 binding model fit was done using Sierra Analyser (Bruker). To ensure the binding properties, the experiments were repeated in an opposite format, where the His‐tagged Fabs were immobilized to a Ni‐NTA sensor chip and the protein Gs were run as analytes. The order of immobilization was reversed when the GA1 avidity was tested (Figure S6). Fab was immobilized via a 6x His‐tag to a Ni‐NTA sensor chip, while mono‐, bi‐, and tri‐valent GA1 constructs were run as analytes.

### 4.8 Fab^H^ ‐GF and GD crystallization and structure determination

Recombinant Fab^H^ E12, its target ASF1 (Bailey et al. 2018) and protein G were produced and purified as described above. SNAP‐tag was removed from the protein G by thrombin‐cleavage at room temperature overnight and the protein G was purified by IMAC on a Talon resin (TaKaRa, Cat # 635653). To obtain the ASF1‐Fab^H^‐protein GF or GD, the proteins were incubated in a 1:1 molar ratio and the complex was purified on a size exclusion chromatography on a Sephadex 200 column, equilibrated with HBS. The purity of the complex was confirmed by SDS‐PAGE. Both protein complexes were concentrated to 10 mg/mL before initial crystallization trials set up at room temperature using the hanging‐drop vapor‐diffusion method utilizing the Mosquito Crystal robot (TTP Labtech).

The ASF1‐Fab^H^E12‐GF complex was crystallized by mixing 100 nL of protein complex solution with 100 nL of a Protein Complex Suite (NeXtal, Cat # 130715) screen solution. The most promising crystals were observed in 0.1 M Sodium Cacodylate pH 5.5, 0.1M Calcium acetate hydrate and 12% PEG 8000. Crystal quality was improved by condition optimization and seeding. Hanging‐drop crystallization was set up by mixing 1 μl of a complex with 1 μl of reservoir solution. Quality was further improved by the seeding technique (Luft and DeTitta 1999) in 0.1M Sodium Cacodylate pH 6.0, 0.1M Calcium acetate hydrate and 10% PEG 8000 at room temperature. The crystals were soaked in mother liquid containing 20% PEG 400 as a cryoprotectant and flash‐frozen in liquid nitrogen for data collection.

The ASF1‐Fab^H^E12‐GD complex was crystallized by mixing 100 nL of protein complex solution with 100 nL of a JCSG Top96 (Rigaku, Cat # 1009846) screen solution. The most promising crystals were observed in 0.1M sodium cacodylate pH 6.5 and 1M sodium citrate tribasic. Crystal quality was improved by condition optimization and seeding. Hanging‐drop crystallization was set up by mixing 1 μl of a complex with 1 μl of reservoir solution. Quality was further improved by the seeding technique (Luft and DeTitta 1999) in 0.1M sodium cacodylate pH 6.8 and 0.8M sodium citrate tribasic at room temperature. The crystals were soaked in mother liquid containing 20% PEG 400 as a cryoprotectant and flash‐frozen in liquid nitrogen for data collection.

X‐ray diffraction datasets were collected at the NECAT 24‐ID‐E beamline at the Advanced Photon Source. Crystal structures were determined by molecular replacement method using the structures of the Fab‐ASF1 complex (pdb: 6AYZ) and protein G (pdb: 6U8C) using PHASER (McCoy et al. 2007). The structure refinements were done using phenix.refine software (Afonine et al. 2012). The electron density maps and the manual corrections were performed using COOT (Emsley and Cowtan 2004). Structural figures were created using ChimeraX (Goddard et al. 2018). Coordinates have been deposited to the Protein Data Bank under the entry: 9AWE and 9AVO.

### 4.9 Flow cytometry

SNAP‐GA1 and SNAP‐GLM were conjugated with BG‐Alexa Fluor 647 and BG‐Alexa Fluor 488 (New England Biolabs, Cat # S9136S, S9129S) respectively, according to the manufacturer's protocol. Excess substrate was removed with PD MiniTrap G‐25 (Cytiva, Cat # 28918007) desalting columns.

A human embryonic kidney (HEK) cell line that was stably transfected to express extracellular MBP anchored to a transmembrane domain and intracellular green fluorescent protein (HEK^M‐^™^‐G^) was used for initial assessment of SNAP‐GA1‐A647 as a secondary detection reagent. HEK^M‐^™^‐G^ cells were cultured to ~70% confluency before detachment by trypsin digestion. Cells were washed once in PBS/1% bovine serum albumin (BSA) and added at a concentration of 500,000 cells per tube to Eppendorf tubes. Cells were incubated with no Fab, 7O^LRT^, 7O^LRT^ in 10 mM maltose, and MJ6^LRT^ (isotype control against Ebola Nucleoprotein) for 30 min at room temperature before washing two times in 1 mL PBS/1% BSA. Next, Alexa Fluor 647 AffiniPure Goat Anti‐Human IgG F(ab′)_2_ fragment specific (Jackson ImmunoResearch, Cat # 109‐036‐003) or SNAP‐GA1‐A647 were added to samples for 20 min at room temperature. Cells were resuspended in 250 μL PBS/1% BSA after the final wash and subjected to analysis by a CytoFLEX flow cytometer (Beckman Coulter).

The human breast cancer cell line SKBR3 (ATCC, Cat # HTB‐30) was cultured to ~70% confluency before detachment by trypsin digestion. Cells were washed once in PBS/1% bovine serum albumin (BSA) and added at a concentration of 500,000 cells per well to a 96‐well round bottom plate. SNAP‐GLM‐A488 and SNAP‐GA1‐A647 were incubated at equimolar concentrations with combinations of MJ20^H^ (isotype control), HER2^H^, 7O^LRT^ (isotype control), and anti‐EGFR^LRT^ respectively, for 1 h on ice before diluting to a final concentration of 250 nM for each component in PBS/1% BSA. Samples were incubated on cells for 30 minutes at room temperature before washing three times with 250 μL of PBS/1% BSA by centrifugation at 400*g* for 5 min. Cells were resuspended in 250 μL PBS/1% BSA after the final wash and subjected to analysis by a CytoFLEX flow cytometer (Beckman Coulter).

### 4.10 Co‐staining detection using flow cytometry

Co‐staining detection of GA1‐hFc and GLM‐mFc with HER2^H^ and EGFR^LRT^ Fabs, respectively, was performed with the HCC1954 cell line. PG‐Fc fusions were pre‐incubated with HER2^H^, EGFR^LRT^, MJ20^H^ (isotype control), or S1^LRT^ (isotype control) on ice for 30 min. The EGFR^LRT^ + GA1‐hFc assembly was first added to cells at 20 nM for 15 min on ice. Cells were washed three times with PBS/1% BSA before adding HER2^H^ + GLM‐mFc at 20 nM for 15 min on ice. Cells were washed three times before adding a mixture of Alexa Fluor 488‐conjugated Anti‐Human Fc (Thermo Scientific, Cat # H10120) and Alexa Fluor 647‐conjugated Anti‐Mouse Fc (Jackson ImmunoResearch, Cat # 115‐605‐205) for 15 min on ice followed by three washes.

### 4.11 Co‐staining detection using microscopy

Hela cells (ATCC, Cat # CCL‐2) were trypsinized and resuspended in DMEM medium (Gibco, Cat # 11965092), including 10% FBS, and then seeded in a cell culture chamber slide (Ibidi, Cat # 80841) at a density of 1.8 × 10^4^ cells/well following incubation in 37°C/5% CO_2_ for 24 h. Cells was treated with 100 nM anti‐EGFR Fab^LRT^ in culture medium for 15 min in 37°C/5% CO_2_, and incubated with 100 nM GA1‐Alexa 647 in 4°C for 1 h after being washed by PBS. Then cells were sequentially fixed by 4% PFA/PBS (Thermo Fisher, Cat # J61899.AP) at RT for 15 min, permeabilized by 0.2%Triton X‐100/PBS (Thermo Fisher, Cat # A16046.AP) at RT for 20 min and blocked by 3% BSA/PBS at RT for 1 h. Lamin A/C Polyclonal antibody (Proteintech, Cat # 10298‐1‐AP) was diluted in 100 nM by 0.5% BSA/PBS and added to cells (150 μL/well) prior to incubation in 4°C overnight. Cells were washed and incubated with 100 nM PG‐Fc‐Alexa 488 in 4°C for 1 h. After being stained by DAPI (Thermo Fisher, Cat # D1306), cells were examined by a confocal microscope (Leica).

### 4.12 Avidity effect testing by the SPR

A multivalent form of protein GA1 was created by introducing a Gly‐Ser linker. Protein GA1 was multimerized from dimer up to decamer with the spectrum of different linkers. The proteins were expressed and purified as described above. His‐tagged Fab in Fab^s^ scaffold was immobilized on the Ni‐NTA sensor chip. Monomer, dimer, and trimer GA1 in two‐fold dilutions starting at 200 nM were run as analytes at 30 μl/min flow rate at 20°C. Sensogram were corrected through double referencing and a 1:1 binding model fit was done using Sierra Analyser (Bruker).

### 4.13 T‐cell redirection cell‐culture assay

Human prostate cancer cell line PC‐3 (ATCC, Cat # CRL‐1435), overexpressing EGFR on the cell surface was cultured according to ATCC protocols. CD3‐positive PBMC cells were isolated from blood (Vissers et al. 1988). The day before the experiment, PC‐3 cells were seeded into a 96‐well plate (20K PC‐3 cells in 100 μl per well). The next day, PBMC cells were added to medium aspirated PC‐3 cells at 10:1 Effector cell to Target cell ratio and then the bi‐specific components were added at 200 nM. After 24 h of co‐culturing, the medium was analyzed for LDH presence using a commercially available kit (CytoTox96, Promega, Cat # G1780). The results were analyzed and normalized using protocols and standards provided in the kit.

### 4.14 Figures generations

The models in the figures were generated using Biorender.com.

## AUTHOR CONTRIBUTIONS

**Tomasz Slezak:** Conceptualization; investigation; writing – original draft; writing – review and editing; visualization; methodology; validation; formal analysis; project administration; supervision. **Kelly M. O'Leary:** Investigation; conceptualization; methodology. **Jinyang Li:** Investigation. **Ahmed Rohaim:** Methodology. **Elena K. Davydova:** Conceptualization. **Anthony A. Kossiakoff:** Conceptualization; methodology; supervision; funding acquisition; writing – original draft; writing – review and editing; project administration.

## Supporting information


**Data S1.** Supporting Information.

## References

[pro70019-bib-0001] Afonine PV , Grosse‐Kunstleve RW , Echols N , Headd JJ , Moriarty NW , Mustyakimov M , et al. Towards automated crystallographic structure refinement with Phenix.Refine. Acta Crystallogr D Biol Crystallogr. 2012;68(Pt 4):352–367. 10.1107/S0907444912001308 22505256 PMC3322595

[pro70019-bib-0002] Agarwal P , Bertozzi CR . Site‐specific antibody‐drug conjugates: the nexus of bioorthogonal chemistry, protein engineering, and drug development. Bioconjug Chem. 2015;26(2):176–192. 10.1021/bc5004982 25494884 PMC4335810

[pro70019-bib-0003] Bailey LJ , Sheehy KM , Dominik PK , Liang WG , Rui H , Clark M , et al. Locking the elbow: improved antibody fab fragments as chaperones for structure determination. J Mol Biol. 2018;430(3):337–347. 10.1016/j.jmb.2017.12.012 29273204 PMC5800945

[pro70019-bib-0004] Bailey LJ , Sheehy KM , Hoey RJ , Schaefer ZP , Ura M , Kossiakoff AA . Applications for an engineered protein‐G variant with a pH controllable affinity to antibody fragments. J Immunol Methods. 2014;415(December):24–30. 10.1016/j.jim.2014.10.003 25450256 PMC4257880

[pro70019-bib-0005] Boder ET , Wittrup KD . Yeast surface display for screening combinatorial polypeptide libraries. Nat Biotechnol. 1997;15(6):553–557. 10.1038/nbt0697-553 9181578

[pro70019-bib-0006] Bradbury ARM , Sidhu S , Dübel S , McCafferty J . Beyond natural antibodies: the power of in vitro display technologies. Nat Biotechnol. 2011;29(3):245–254. 10.1038/nbt.1791 21390033 PMC3057417

[pro70019-bib-0007] Couchman JR . Commercial antibodies: the good, bad, and really ugly. J Histochem Cytochem. 2009;57(1):7–8. 10.1369/jhc.2008.952820 18854593 PMC2605718

[pro70019-bib-0008] Emsley P , Cowtan K . Coot: model‐building tools for molecular graphics. Acta Crystallogr D Biol Crystallogr. 2004;60(Pt 12):2126–2132. 10.1107/S0907444904019158 15572765

[pro70019-bib-0009] Goddard TD , Huang CC , Meng EC , Pettersen EF , Couch GS , Morris JH , et al. UCSF ChimeraX: meeting modern challenges in visualization and analysis. Protein Sci. 2018;27(1):14–25. 10.1002/pro.3235 28710774 PMC5734306

[pro70019-bib-0010] Köhler G , Milstein C . Continuous cultures of fused cells secreting antibody of predefined specificity. Nature. 1975;256(5517):495–497. 10.1038/256495a0 1172191

[pro70019-bib-0011] Kunkel TA . Rapid and efficient site‐specific mutagenesis without phenotypic selection. Proc Natl Acad Sci U S A. 1985;82(2):488–492. 10.1073/pnas.82.2.488 3881765 PMC397064

[pro70019-bib-0012] Lan T , Slezak T , Jinyue P , Zinkus‐Boltz J , Adhikari S , Pekow JR , et al. Development of luminescent biosensors for calprotectin. ACS Chem Biol. 2024;19(6):1250–1259. 10.1021/acschembio.4c00265 38843544 PMC12072359

[pro70019-bib-0013] Luft JR , DeTitta GT . A method to produce microseed stock for use in the crystallization of biological macromolecules. Acta Crystallogr D Biol Crystallogr. 1999;55(Pt 5):988–993. 10.1107/s0907444999002085 10216295

[pro70019-bib-0014] McCoy AJ , Grosse‐Kunstleve RW , Adams PD , Winn MD , Storoni LC , Read RJ . Phaser crystallographic software. J Appl Cryst. 2007;40(Pt 4):658–674. 10.1107/S0021889807021206 19461840 PMC2483472

[pro70019-bib-0022] Sauer‐Eriksson AE, Kleywegt GJ, Uhlén M, Jones TA . Crystal structure of the C2 fragment of streptococcal protein G in complex with the Fc domain of human IgG. Struct. 1995;3(3):265–278. 10.1016/s0969-2126(01)00157-5 7788293

[pro70019-bib-0015] Schumacher S , Seitz H . Quality control of antibodies for assay development. N Biotechnol. 2016;33:544–550. 10.1016/j.nbt.2016.02.001 26873787

[pro70019-bib-0016] Sidhu SS , Lowman HB , Cunningham BC , Wells JA . Phage display for selection of novel binding peptides. Methods Enzymol. 2000;328:333–363. 10.1016/s0076-6879(00)28406-1 11075354

[pro70019-bib-0017] Slezak T , Bailey LJ , Jaskolowski M , Nahotko DA , Filippova EV , Davydova EK , et al. An engineered ultra‐high affinity fab‐protein G pair enables a modular antibody platform with multifunctional capability. Protein Sci. 2020;29(1):141–156. 10.1002/pro.3751 31622515 PMC6933859

[pro70019-bib-0018] Slezak T , Kossiakoff AA . Engineered ultra‐high affinity synthetic antibodies for SARS‐CoV‐2 neutralization and detection. J Mol Biol. 2021;433(10):166956. 10.1016/j.jmb.2021.166956 33775667 PMC7997149

[pro70019-bib-0019] Smith GP . Filamentous fusion phage: novel expression vectors that display cloned antigens on the Virion surface. Science (New York, NY). 1985;228(4705):1315–1317. 10.1126/science.4001944 4001944

[pro70019-bib-0020] Vissers MC , Jester SA , Fantone JC . Rapid purification of human peripheral blood monocytes by centrifugation through Ficoll‐Hypaque and Sepracell‐MN. J Immunol Methods. 1988;110(2):203–207. 10.1016/0022-1759(88)90104-4 2837516

[pro70019-bib-0021] Viti F , Nilsson F , Demartis S , Huber A , Neri D . Design and use of phage display libraries for the selection of antibodies and enzymes. Methods Enzymol. 2000;326:480–505. 10.1016/s0076-6879(00)26071-0 11036659

[pro70019-bib-0023] Von Kreudenstein TS, Escobar‐Carbrera E, Lario PI, D’Angelo I, Brault K, Kelly J , et al. Improving biophysical properties of a bispecific antibody scaffold to aid developability: quality by molecular design. mAbs. 2013;5(5):646–654. 10.4161/mabs.25632 23924797 PMC3851217

