# Supplementary material for: Engineered protein G variants for multifunctional antibody‐based assemblies
Source: Protein Sci. 2025 Jan 25;34(2):e70019. doi: 10.1002/pro.70019 (PMC11761708; doi:10.1002/pro.70019)
Supplement: Supplementary file 1 — Data S1. Supporting Information. [file PRO-34-e70019-s001.docx]

**Engineered Protein G Variants for Multifunctional Antibody-Based Assemblies**

Tomasz Slezak^1^, Kelly M. O’Leary^1^, Jinyang Li^1^, Ahmed Rohaim^1^, Elena K. Davydova^1^, Anthony A. Kossiakoff^1,2^

1. Department of Biochemistry and Molecular Biology, The University of Chicago, Chicago, Illinois.
2. Institute for Biophysical Dynamics, The University of Chicago, Chicago, Illinois.

*Correspondence to: Anthony A. Kossiakoff, The University of Chicago, 900 East 57^th^ Street, Chicago, IL 60637, koss@bsd.uchicago.edu

**Supplementary Materials**

**
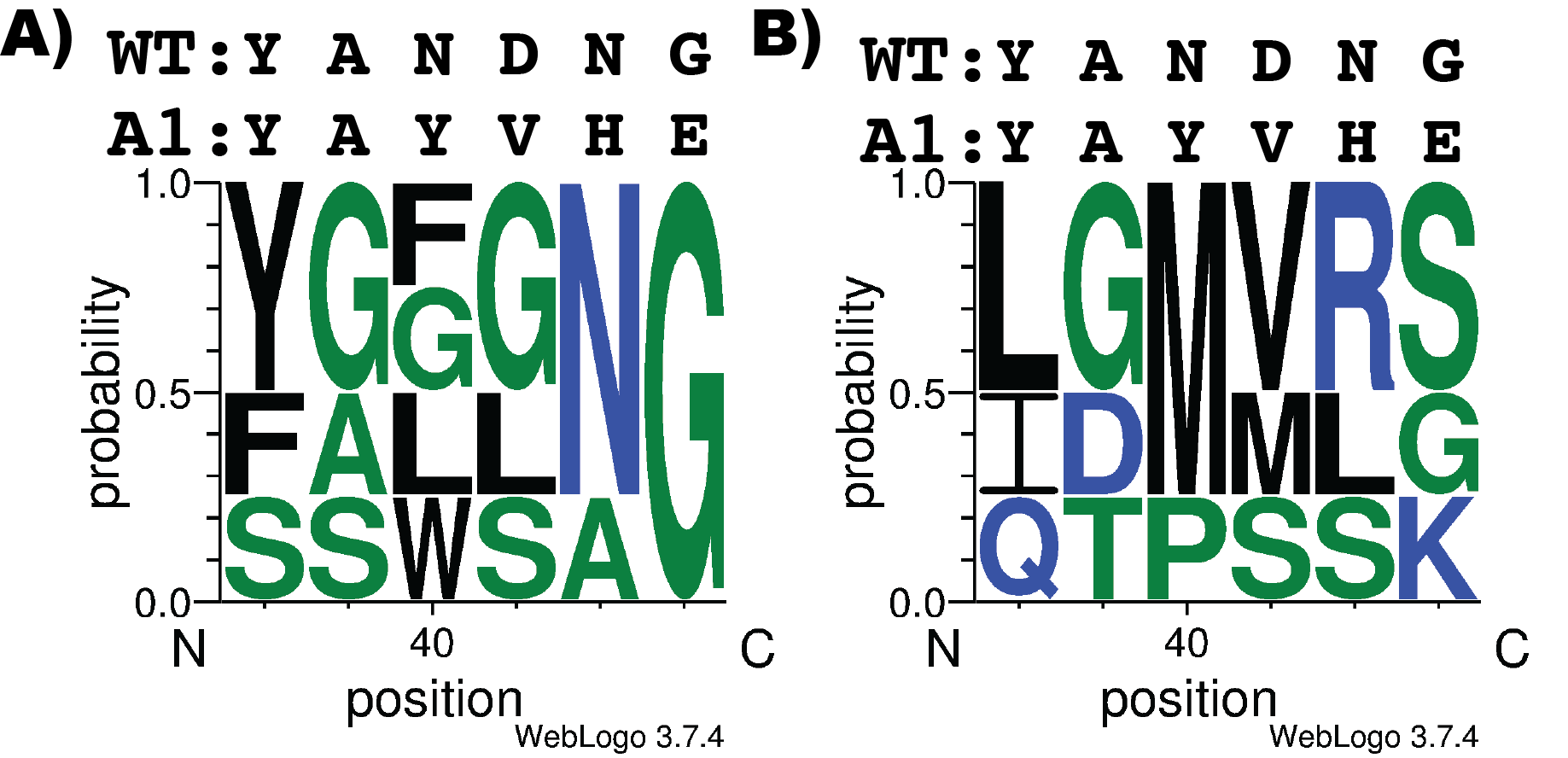
**

**Figure S1:** (**A**) Weblogo represents amino acids sequences of newly developed protein Gs that pose universal binding properties (binds all existing Fab scaffolds). (**B**) Weblogo represents protein Gs selectively binding only Fab^H^ and Fab^L^, but do not bind to Fab^LRT^. The sequences corresponding to the 38-43 positions of wt PG and GA1 are shown on top.

**
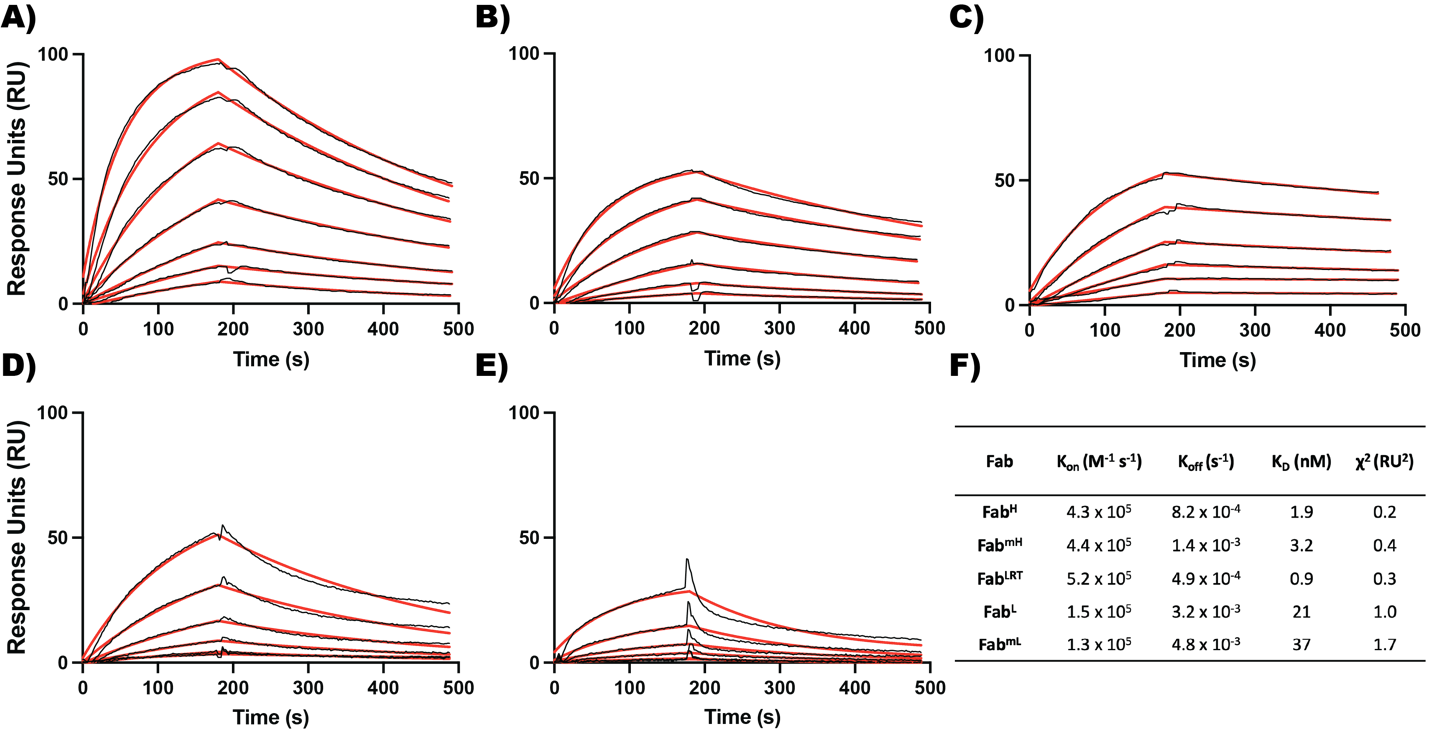
**

**Figure S2: Characterization of GF- an universal Fab binder.** Protein GF was immobilized on the NTA Chip. (A) SPR sensogram showing the interaction with Fab^H^. (B) SPR sensogram showing the intraction with Fab^mH^. (C) SPR sensogram showing the interaction with Fab^LRT^. (D) SPR sensogram showing the interaction with Fab^L^. (E) SPR sensogram showing the interaction with Fab^mL^. (F) Kinetic binding parameters. For the kinetic experiment, Fabs were serially diluted two-fold, starting at 50 nM for all Fabs, and 25 nM for Fab^LRT^. SPR experiments were performed at room temperature.

**
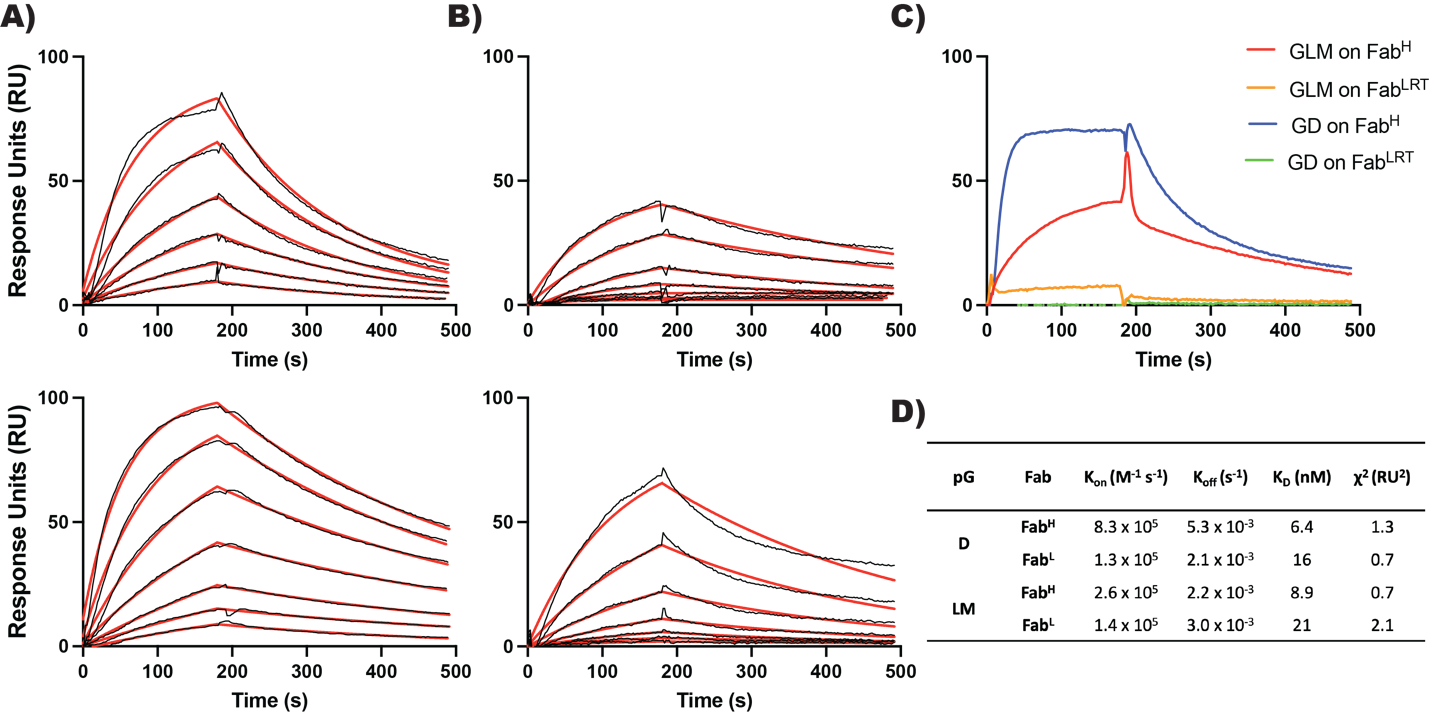
**

**Figure S3: Characterization of GD and GLM.** Protein GLM or GD were immobilized on the NTA Chip. (A) SPR sensogram showing the interaction of GD (top) and GLM (bottom) with Fab^H^. (B) SPR sensogram showing the interaction of GD (top) and GLM (bottom) with Fab^L^. (C) A single injection of Fab^H^ and Fab^LRT^. 25nM of each Fab was injected, and no binding to Fab^LRT^ was observed. (D) Kinetic parameters of binding. For the kinetic experiment, Fabs were serially diluted two-fold, starting at 100nM. SPR experiments were performed at room temperature.


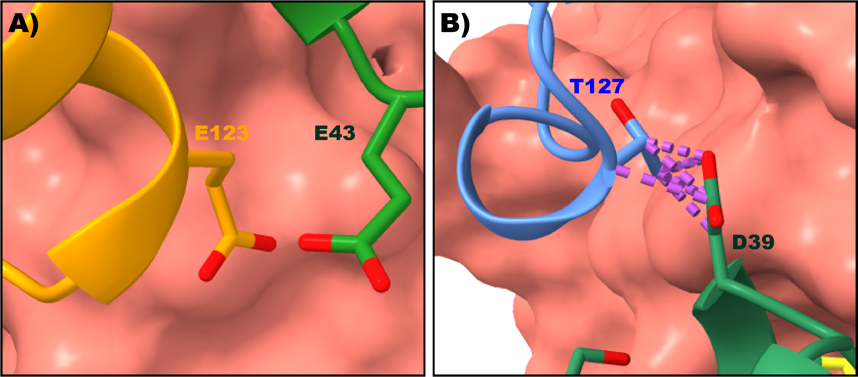


**Figure S4: Structural basis of an orthogonal specificity between GA1-Fab^LRT^ and GD-Fab^H^.** (A) Model of GA1-Fab^H^. The charge clash of E123 from Fab^H^ and E43 from GA1 is responsible for no interaction between these molecules. (B) Model of GD-Fab^LRT^. Light chain loop rearrangement caused by two amino acids deletion in Fab^LRT^ causes a clash between T127 and D39 from the GD. Molecules are colored as follows: Fab Hc- red, Fab^H^ Lc- orange, Fab^LRT^ Lc- blue, GA1, and GD- green.

**Figure S5:** **Stability of Plug and Play PG complexes in cell-based applications.** The durability of the interaction between GLM and Fab^H^ was assessed using flow cytometry. In this assay, Hela cells were initially labeled with EGFR Fab^H^, followed by the application of fluorescently-tagged GLM-Alexa647 as a secondary detection reagent. This methodology allowed for the visualization and quantification of the complex stability under physiological conditions for 24 hours.

**
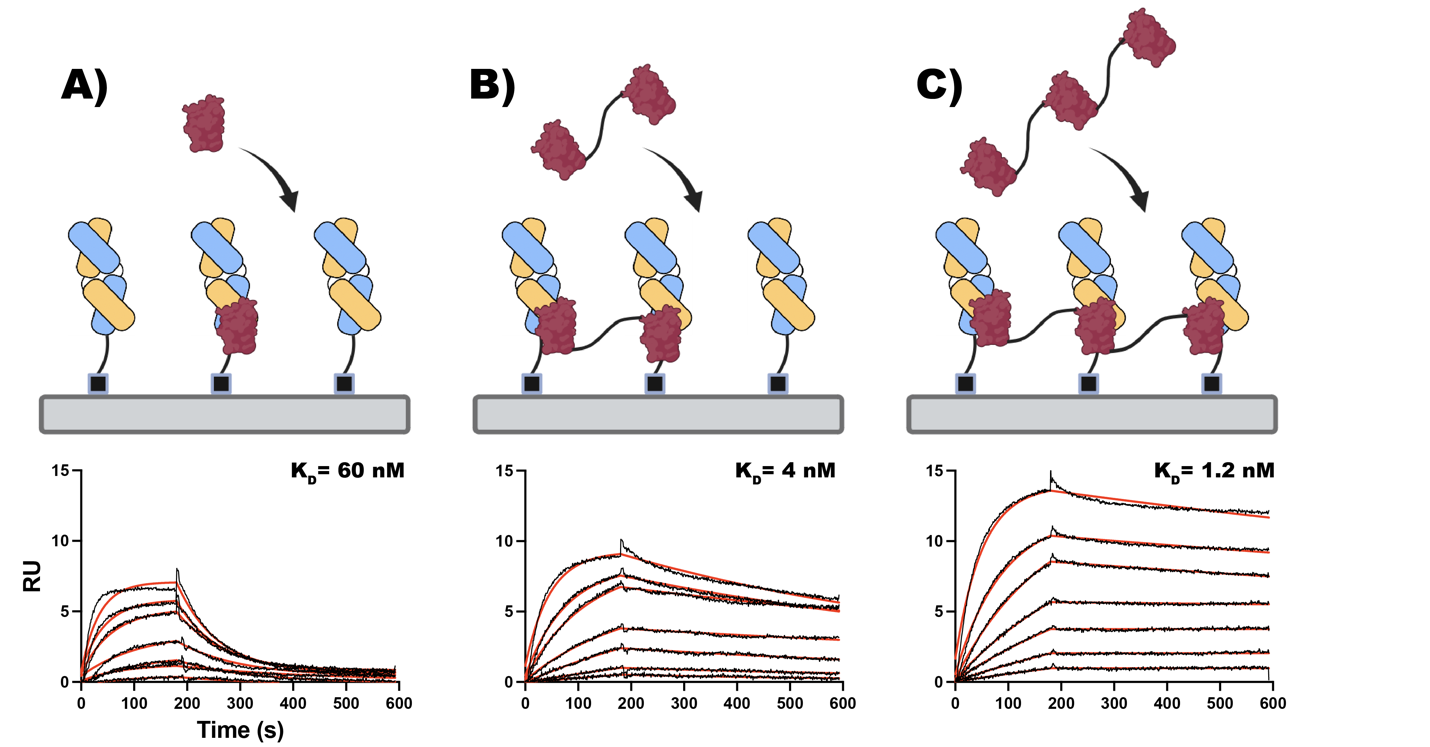
**

**Figure S6: Avidity-driven GA1 dissociation rate enhancement.** (A) SPR sensogram showing the interaction with GA1 monomer and Fab^S^. (B) SPR sensogram showing the interaction with GA1 dimer and Fab^S^. (C) SPR sensogram showing the interaction with GA1 trimer and Fab^S^. The kinetic parameters exhibit substantially decreased k_off_ values with increasing GA1 valency but only marginally decreased k_on_ values. SPR experiments were performed at room temperature.


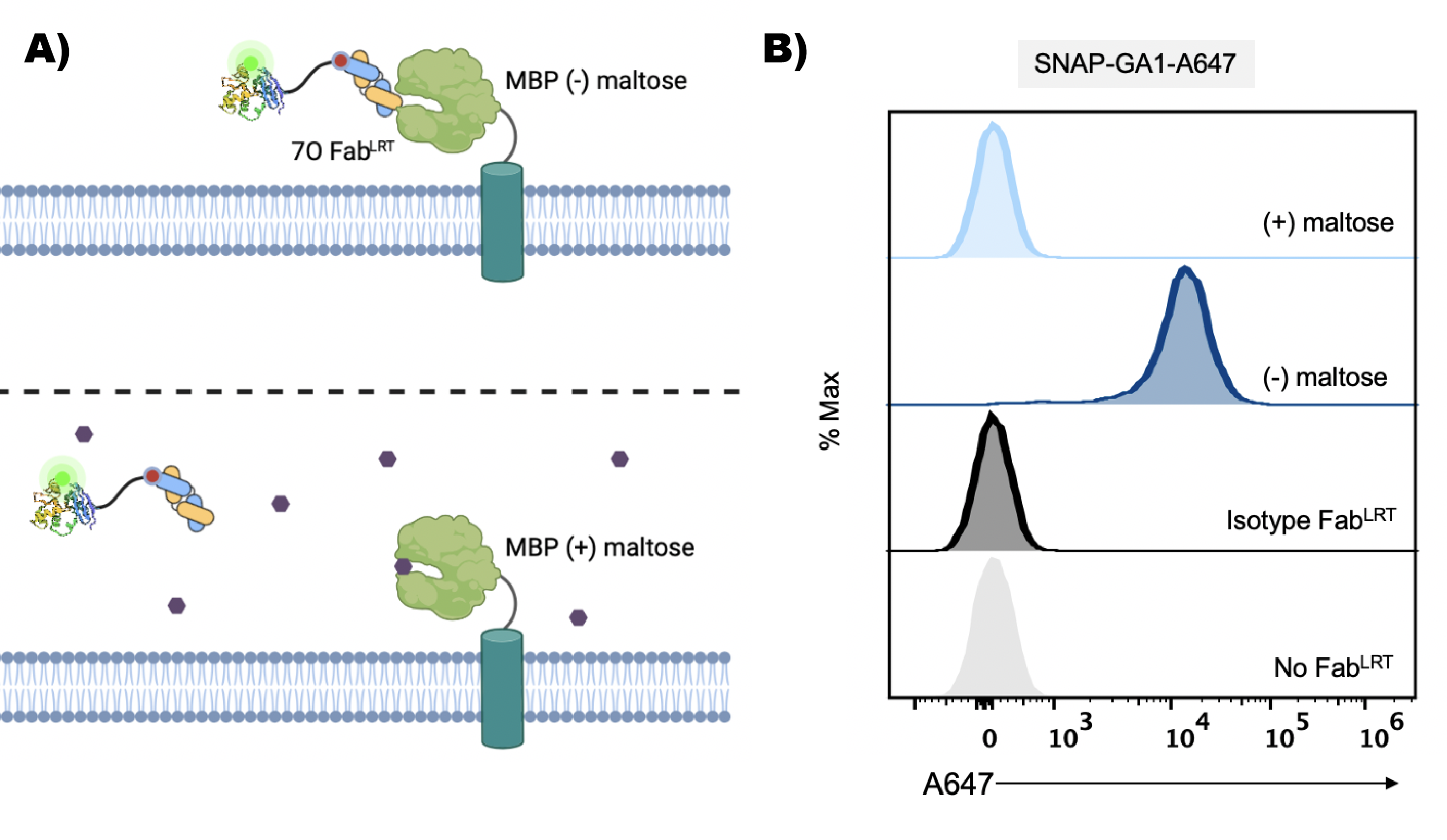


**Figure S7: Labeled SNAP-protein G as a tool for antibody binding detection by flow cytometry**. (**A**) Experimental model. A conformation specific anti-MBP 7O Fab^LRT^ was used to detect the extracellular MBP stably engineered on the surface of the HEK cell line. Conformational change of MBP upon maltose addition eliminates the anti-MBP 7O Fab^LRT^ binding. SNAP-GA1 labeled with Alexa 647 is used for the detection in flow cytometry and is premixed with Fab before the cell staining. (**B**) Flow cytometry histogram of anti-MBP 7O Fab^LRT^ binding to HEK cell line stably expressing extracellular MBP without the presence of the maltose. The system exhibits a strong signal with a shallow background. Fab binding is abolished upon maltose addition. As a negative control the SNAP-GA1 alone and the isotype Fab against Ebola nucleoprotein were used. No detectable signal was observed. Fab is premixed with SNAP-GA1 before cell staining, which significantly shortens the staining protocol.


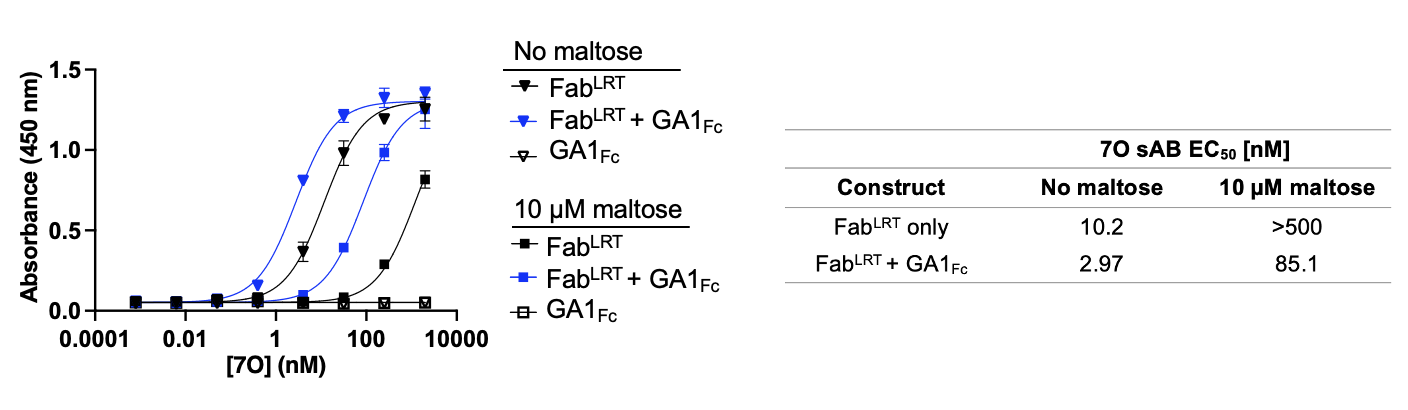


**Figure S8: GA1_Fc_ fusion enables modular assembly of bivalent IgG-like assemblies.** (**A**) ELISA EC_50_ analysis of Fab 7O binding to immobilized MBP in the absence of maltose or with 10 µM maltose. In both cases, the IgG-like assembly format improves the 7O EC_50_ value substantially. (**B**) Table of 7O EC_50_ values based on the concentration of Fab used for the experiment.


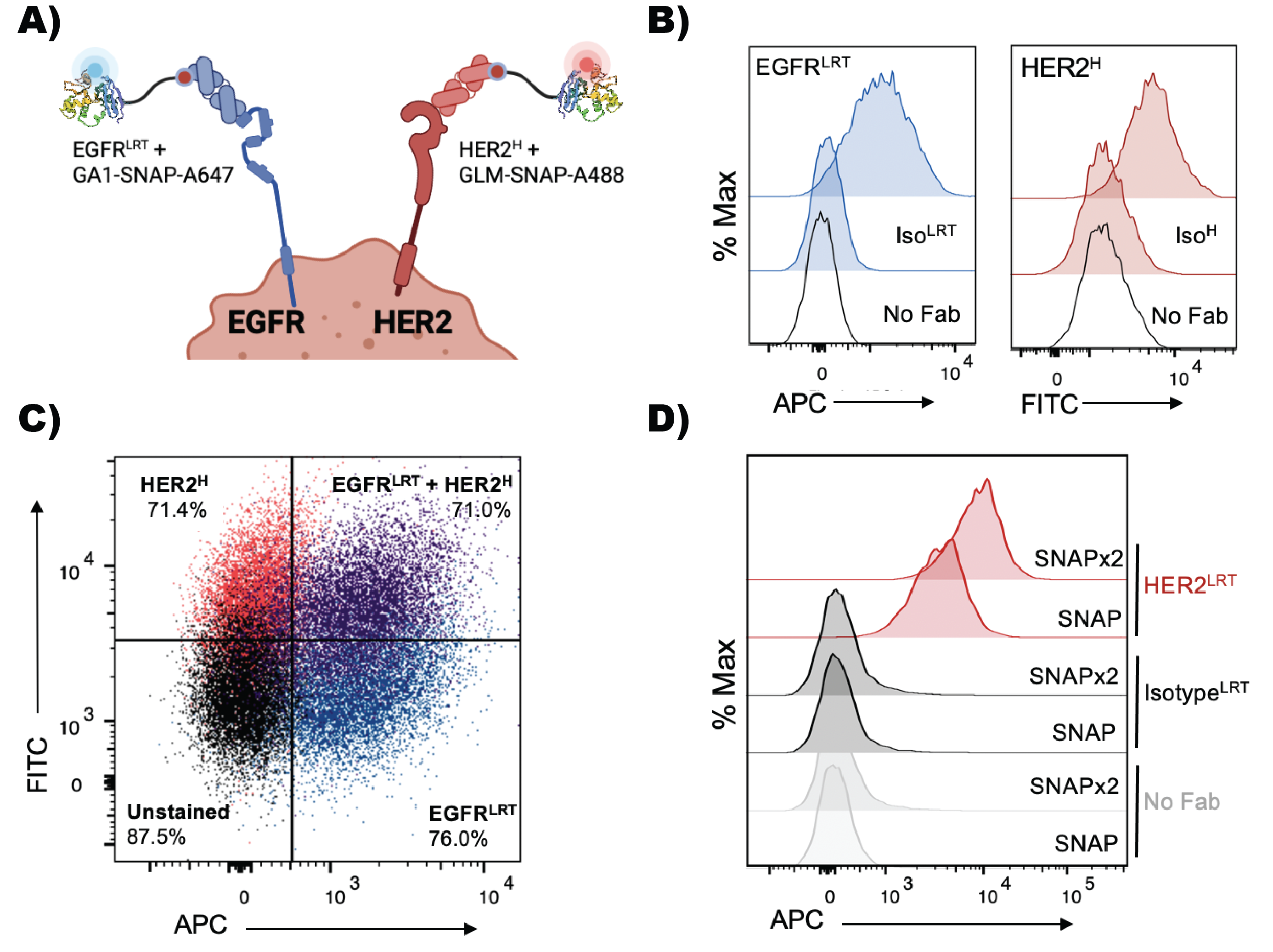


**Figure S9: Flow cytometry simultaneous binding detection of two different Fabs by the orthogonal pairing of GA1-Fab^LRT^ and GLM-Fab^H^. (A)** Model of secondary co-detection using the orthogonal pairing of GA1-Fab^LRT^ and GLM-Fab^H^. SNAP-GA1 and SNAP-GLM fusions are labeled with benzylguanine- (BG) Alexa 647 and Alexa 488, respectively, and then incubated with Fab^LRT^ and Fab^H^ molecules before addition to cells. Protein GA1 and GLM scaffold specificity allow for the simultaneous detection of the other Fab binding on the cell surface. **(B)** Flow cytometry analysis of SKBR3 cell surface receptors. EGFR was detected by an anti-EGFR Fab^LRT^ using SNAP-GA1-A647 as a secondary detection agent (*left*). HER2 was detected by an anti-HER2 Fab^H^ using SNAP-GLM-A488 as a secondary detection agent (*right*). **(C)**Density plot analysis of**s**imultaneous detection of anti-EGFR Fab^LRT^ and anti-HER2 Fab^H^ via flow cytometry using SNAP-GA1-A647 and SNAP-GLM-A488, respectively, as secondary detection agents. The high degree of specificity for each GA1-Fab^LRT^and GLM-Fab^H^ interactions allows all Fab and secondary detection components to be mixed in one tube before adding to cells. The low background and depleted off-target recognition are demonstrated using Fab^LRT^ and Fab^H^ isotype controls. **(D)** Double SNAP fusion significantly increased detected signal.


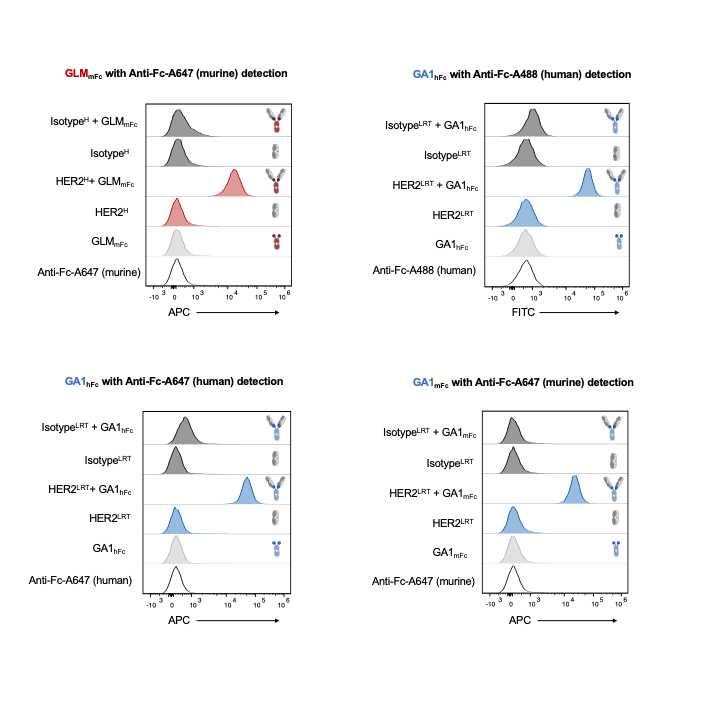


**Figure S10: Cell surface staining controls for the modular assembly of protein G_Fc_ fusions with HER2 targeting Fabs.** MDA-MB-453 cells were stained with IgG-like assembled sABs targeting EGFR (Fab^LRT^ format) or HER2 (Fab^H^ format) at 200 nM. No background signal was detected using Fc-fusion only, Fab only, or the isotype controls.


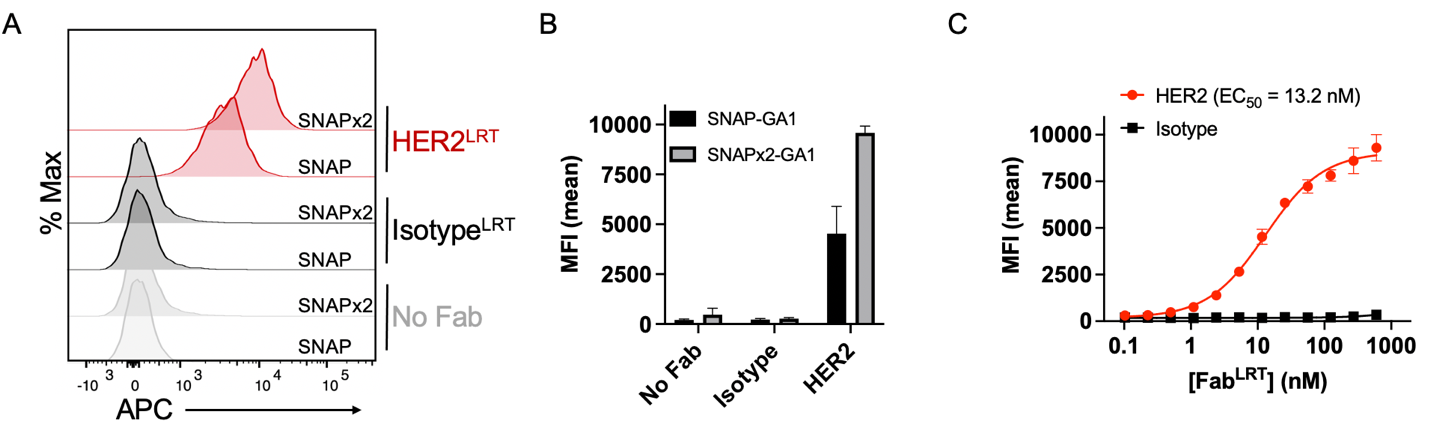


**Figure S11: GA1 fusion to tandem SNAP-tag enables low wash cell staining protocols.** A) MDA-MB-453 cells were stained with HER2 targeting Fab^LRT^ or isotype control Fab^LRT^ at 200 nM. Fabs were pre-incubated at an equimolar ratio with SNAP-GA1 or SNAPx2-GA1 labeled with Alexa Fluor 647. B) MFI quantification of the results in A shows that two SNAP-tags labeled with Alexa Fluor 647 doubles the signal. C) Cell surface EC_50_ using 50 nM SNAPx2-GA1 labeled with Alexa Fluor 647 as a secondary detection agent.

**
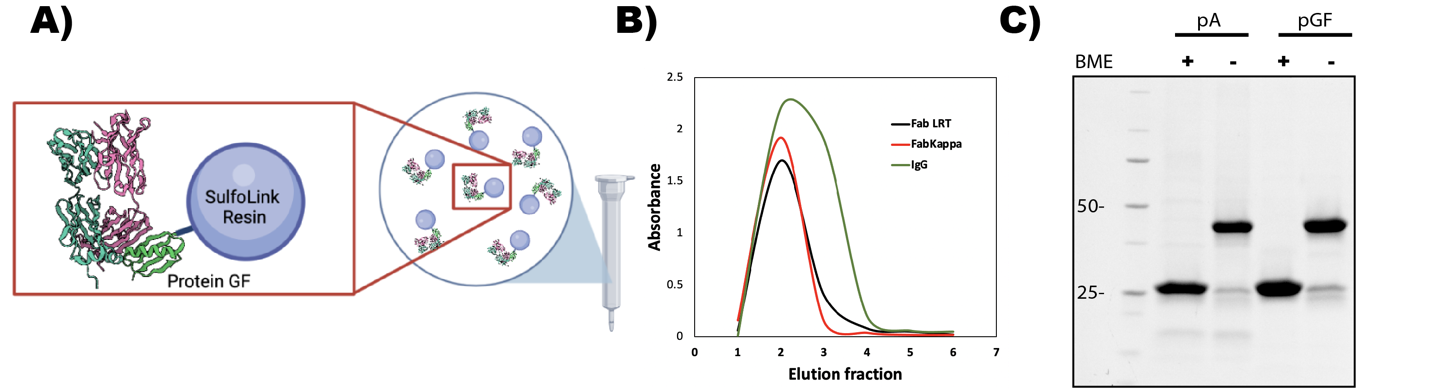
**

**Figure S12: In-house generation of affinity chromatography resin for universal Fab purification.** (A) Schematic showing the covalent immobilization of SUMO-GF with SulfoLink Coupling Resin. The resin is a high capacity (20mg of Fab/mL), has a long lifespan (>1.5 years), and is reusable due to its tolerance to low pH regeneration. (B) Elution profiles of full-length IgG, Fab^H^, and Fab^LRT^ purified by a single step with a SUMO-GF coupled resin. (C) SDS-PAGE gel post purification. Purity of the product is superior to the commonly used Protein-A resin due to its single chain binding profile.

**Figure S13. Efficiency of Plug-and-Play Bispecific T-cell Engager (BiTE).** The Her2 Fab^LRT^, which recognizes the HER2 extracellular domain on antigen-presenting cells (APCs), was preincubated with GA1-OKT3, which binds to the CD3 molecule on T-cells, to form a plug-and-play BiTE. The efficacy of this BiTE was evaluated in a PBMC/PC3 (10:1) co-culture system. Cytotoxicity of cancer cells was assessed using an LDH release assay after 24 hours of incubation. The efficiency of the BiTE, as measured by its EC50 value, corresponds closely to the K_D_ value (0.1 nM) of the GA1-Fab^LRT^ interaction. This correlation demonstrates that the functionality of plug-and-play BiTEs is fundamentally governed by the thermodynamic properties of the system.


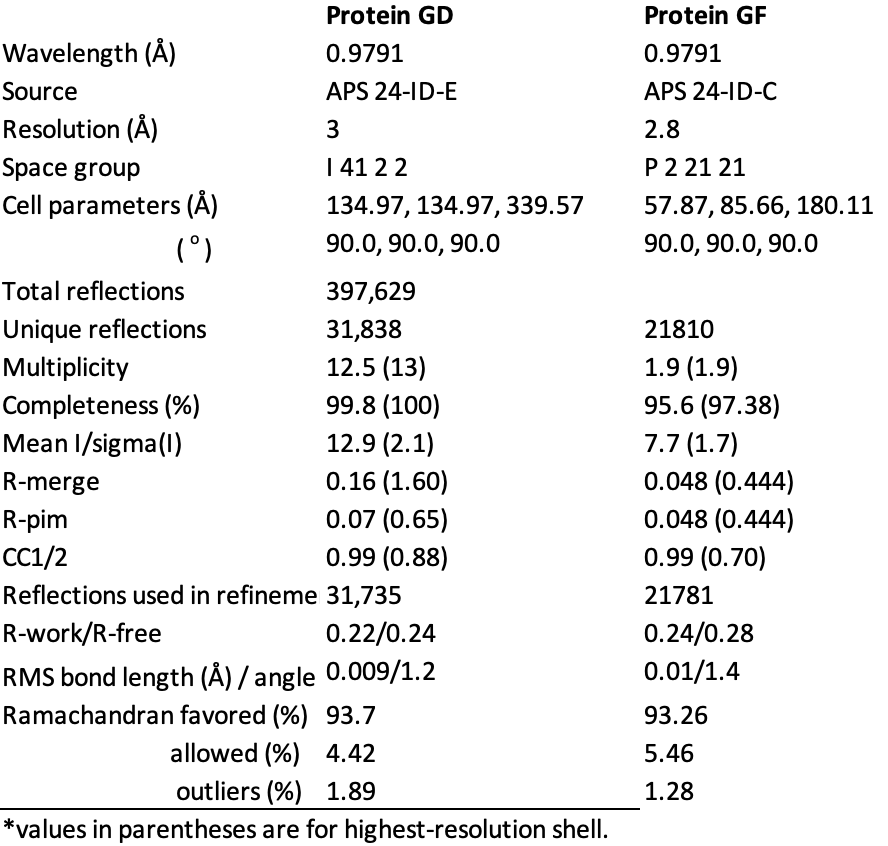


**Table S1. Data collection and refinement statistics summary**

| **Protein G** | **Amino acid sequence in position 38-43** | **K_on_ (M^-1^ s^-1^)** | **K_off_ (s^-1^)** | **K_D_ (nM)** |
| --- | --- | --- | --- | --- |
| GF | YAFGNG | 4.3 x 10^5^ | 8.2 x 10^-4^ | 1.9 |
| GD | IDMVSS | 8.3 x 10^5^ | 5.3 x 10^-3^ | 6.4 |
| GLM | LGMMRS | 2.6 x 10^5^ | 2.2 x 10^-3^ | 8.9 |
| GS | SGLLAG | 2.8 x 10^5^ | 1.6 x 10^-3^ | 5.8 |
| GLV | LGMVRG | 2.9 x 10^5^ | 4.8 x 10^-3^ | 16.5 |
| GYG | YGTANG | 3.6 x 10^5^ | 2.6 x 10^-3^ | 7.2 |
| GQT | QTPSLK | 2.5 x 10^5^ | 3.2 x 10^-3^ | 13.0 |
| GW | FGWSNG | 2.7 x 10^5^ | 1.2 x 10^-3^ | 4.3 |
| GY | YSGGNG | 3.7 x 10^5^ | 1.4 x 10^-3^ | 3.9 |
| GFH | FAHGNA | 2.0 x 10^5^ | 2.1 x 10^-3^ | 10.4 |
| GFS | FGNSNG | 2.3 x 10^5^ | 1.9 x 10^-3^ | 8.3 |

**Table S2. Kinetic parameters of different Protein GS binding to Fab^H^.**

| **Protein G** | **Amino acid sequence in position 38-43** | **K_on_ (M^-1^ s^-1^)** | **K_off_ (s^-1^)** | **K_D_ (nM)** |
| --- | --- | --- | --- | --- |
| GF | YAFGNG | 5.2 x 10^5^ | 4.9 x 10^-4^ | 0.9 |
| GA1 | YAYVHE | 2.6 x 10^6^ | 2.4 x 10^-4^ | 0.1 |
| GS | SGLLAG | 3.7 x 10^5^ | 2.4 x 10^-3^ | 6.6 |
| GW | FGWSNG | 3.2 x 10^5^ | 1.4 x 10^-3^ | 4.2 |
| GY | YSGGNG | 5.2 x 10^5^ | 1.6 x 10^-3^ | 3.2 |

**Table S3. Kinetic parameters of different Protein G-s binding to Fab^LRT^.**


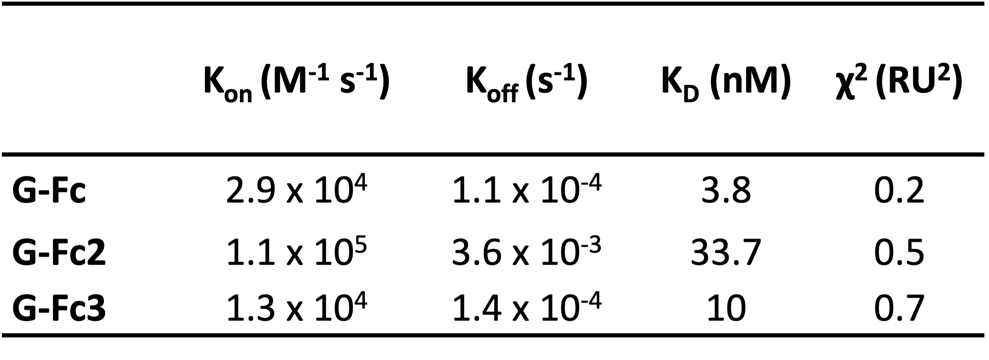


**Table S5.** **Kinetic parameters of Protein G-Fc binding to Human Fc.**

**Table S5. Sequences**

**PGA1_SNAP_H6 (pEKD40)**

MASTMDIKLTGEFAMDKDCEMKRTTLDSPLGKLELSGCEQGLHEIKLLGKGTSAADAVEVPAPAAVLGGPEPLMQATAWLNAYFHQPEAIEEFPVPALHHPVFQQESFTRQVLWKLLKVVKFGEVISYQQLAALAGNPAATAAVKTALSGNPVPILIPCHRVVSSSGAVGGYEGGLAVKEWLLAHEGHRLGKPGLGLVPRGSPAGGSPTPAVTTYKLVINGRTLSGYTTTTAVDAATAEKVFKQYAYVHEVDGEWTYDDATKTFTVTEKPEKLGLEHHHHHH

**PGLM_SNAP_H6 (pEKD40)**

MASTMDIKLTGEFAMDKDCEMKRTTLDSPLGKLELSGCEQGLHEIKLLGKGTSAADAVEVPAPAAVLGGPEPLMQATAWLNAYFHQPEAIEEFPVPALHHPVFQQESFTRQVLWKLLKVVKFGEVISYQQLAALAGNPAATAAVKTALSGNPVPILIPCHRVVSSSGAVGGYEGGLAVKEWLLAHEGHRLGKPGLGLVPRGSPAGGSPTPAVTTYKLVINGRTLSGYTTTTAVDAATAEKVFKQLGMMRSVDGEWTYDDATKTFTVTEKPEKLGLEHHHHHH

**PGD_SNAP_H6 (pEKD40)**

MASTMDIKLTGEFAMDKDCEMKRTTLDSPLGKLELSGCEQGLHEIKLLGKGTSAADAVEVPAPAAVLGGPEPLMQATAWLNAYFHQPEAIEEFPVPALHHPVFQQESFTRQVLWKLLKVVKFGEVISYQQLAALAGNPAATAAVKTALSGNPVPILIPCHRVVSSSGAVGGYEGGLAVKEWLLAHEGHRLGKPGLGLVPRGSPAGGSPTPAVTTYKLVINGRTLSGYTTTTAVDAATAEKVFKQIDMVSSVDGEWTYDDATKTFTVTEKPEKLGLEHHHHHH

**PF_SNAP_H6 (pEKD40)**

MASTMDIKLTGEFAMDKDCEMKRTTLDSPLGKLELSGCEQGLHEIKLLGKGTSAADAVEVPAPAAVLGGPEPLMQATAWLNAYFHQPEAIEEFPVPALHHPVFQQESFTRQVLWKLLKVVKFGEVISYQQLAALAGNPAATAAVKTALSGNPVPILIPCHRVVSSSGAVGGYEGGLAVKEWLLAHEGHRLGKPGLGLVPRGSPAGGSPTPAVTTYKLVINGRTLSGYTTTTAVDAATAEKVFKQYAFGNGVDGEWTYDDATKTFTVTEKPEKLGLEHHHHHH

**PGD_H10 (pHFT2)**

MKHHHHHHHHHHSSDYKDDDDKGENLYFQGSTPAVTTYKLVINGRTLSGYTTTTAVDAATAEKVFKQIDMVSSVDGEWTYDDATKTFTVTEKPEKL

**PGLM_H10 (pHFT2)**

MKHHHHHHHHHHSSDYKDDDDKGENLYFQGSTPAVTTYKLVINGRTLSGYTTTTAVDAATAEKVFKQLGMMRSVDGEWTYDDATKTFTVTEKPEKL

**PGF_H10 (pHFT2)**

MKHHHHHHHHHHSSDYKDDDDKGENLYFQGSTPAVTTYKLVINGRTLSGYTTTTAVDAATAEKVFKQYAFGNGVDGEWTYDDATKTFTVTEKPEKL

**PG-FC (pHFT2)**

MKHHHHHHHHHHSSDYKDDDDKGENLYFQGSTPAVTTYKLVINGKTLKGETTTKAVDAETAEKAFKQYANVHEVDGEWTYDDATKTFTVTEKPEKL

**PG-FC2 (pHFT2)**

MKHHHHHHHHHHSSDYKDDDDKGENLYFQGSTPAVTTYKLVINGKTLKGETTTKAVDAETAEKAFKQYAYVHEVDGEWTYDDATKTFTVTEKPEKL

**PG-FC3 (pHFT2)**

MKHHHHHHHHHHSSDYKDDDDKGENLYFQGSTPAVTTYKLVINGKTLKGETTTKAVDAETAEKAFKQYANDNEVDGEWTYDDATKTFTVTEKPEKL

**ASF1_H10 (pHFT2)**

MKHHHHHHHHHHSSDYKDDDDKGENLYFQGSSSIVSLLGIKVLNNPAKFTDPYEFEITFECLESLKHDLEWKLTYVGSSRSLDHDQELDSILVGPVPVGVNKFVFSADPPSAELIPASELVSVTVILLSCSYDGREFVRVGYYVNNEYDEEELRENPPAKVQVDHIVRNILAEKPRVTRFNIVWDNENEGLE

**Asf1 E11 Fab^H^ (RH2.2)**

SDIQMTQSPSSLSASVGDRVTITCRASQSVSSAVAWYQQKPGKAPKLLIYSASSLYSGVPSRFSGSRSGTDFTLTISSLQPEDFATYYCQQSSDDPITFGQGTKVEIKRTVAAPSVFIFPPSDEQLKSGTASVVCLLNNFYPREAKVQWKVDNALQSGNSQESVTEQDSKDSTYSLSSTLTLSKADYEKHKVYACEVTHQGLSSPVTKSFNRGEC/EISEVQLVESGGGLVQPGGSLRLSCAASGFNISYSSIHWVRQAPGKGLEWVASISSYYGSTYYADSVKGRFTISADTSKNTAYLQMNSLRAEDTAVYYCARSRGQASWDYWGQGTLVTVSSASTKGPSVFPLAPSSKSTSGGTAALGCLVKDYFPEPVTVSWNSGALTSGVHTFPAVLQSSGLYSLSSVVTVPSSSLGTQTYICNVNHKPSNTKVDKKVEPKSCDKTHT

**ASF1 E5 Fab^H^ (RH2.2)**

SDIQMTQSPSSLSASVGDRVTITCRASQSVSSAVAWYQQKPGKAPKLLIYSASSLYSGVPSRFSGSRSGTDFTLTISSLQPEDFATYYCQQDGWSLITFGQGTKVEIKRTVAAPSVFIFPPSDEQLKSGTASVVCLLNNFYPREAKVQWKVDNALQSGNSQESVTEQDSKDSTYSLSSTLTLSKADYEKHKVYACEVTHQGLSSPVTKSFNRGEC**/**EISEVQLVESGGGLVQPGGSLRLSCAASGFNVSYYSIHWVRQAPGKGLEWVASIYPYYGSTSYADSVKGRFTISADTSKNTAYLQMNSLRAEDTAVYYCARGYGWALDYWGQGTLVTVSSASTKGPSVFPLAPSSKSTSGGTAALGCLVKDYFPEPVTVSWNSGALTSGVHTFPAVLQSSGLYSLSSVVTVPSSSLGTQTYICNVNHKPSNTKVDKKVEPKSCDKTHT

**hUCHT1 Fab^LRT^ (RH2.2)**

SDIQMTQSPSSLSASVGDRVTITCSASQDIRNYLNWYQQKPGKAPKRWIYYTSRLHSGVPSRFSGSGSGTDYTLTISSLQPEDFATYYCQQGNTLPWTFGQGTKVEIKRTVAAPSVFIFPPSDLRTGTASVVCLLNNFYPREAKVQWKVDNALQSGNSQESVTEQDSKDSTYSLSSTLTLSKADYEKHKVYACEVTHQGLSSPVTKSFNRGEC/EISEVQLVESGGGLVQPGGSLRLSCAASGFNFTGYTIHWVRQAPGKGLEWMGLINPYKGVSTYNQKFKDKATISTDKSKNTAYLQMNSLRAEDTAVYYCARSGYYGDSDWYFDYWGQGTLVTVSSASTKGPSVFPLAPSSKSTSGGTAALGCLVKDYFPEPVTVSWNSGALTSGVHTFPAVLQSSGLYSLSSVVTVPSSSLGTQTYICNVNHKPSNTKVDKKVEPKSCDKTHT

**11M MBP Fab^LRT^ (RH2.2)**

SDIQMTQSPSSLSASVGDRVTITCRASQSVSSAVAWYQQKPGKAPKLLIYSASSLYSGVPSRFSGSRSGTDFTLTISSLQPEDFATYYCQQASLTALLTFGQGTKVEIKRTVAAPSVFIFPPSDLRTGTASVVCLLNNFYPREAKVQWKVDNALQSGNSQESVTEQDSKDSTYSLSSTLTLSKADYEKHKVYACEVTHQGLSSPVTKSFNRGECEISEVQLVESGGGLVQPGGSLRLSCAASGFNLSSSSIHWVRQAPGKGLEWVASIYSYYGSTSYADSVKGRFTISADTSKNTAYLQMNSLRAEDTAVYYCAREYHSYWSYSWWPRVGLDYWGQGTLVTVSSASTKGPSVFPLAPSSKSTSGGTAALGCLVKDYFPEPVTVSWNSGALTSGVHTFPAVLQSSGLYSLSSVVTVPSSSLGTQTYICNVNHKPSNTKVDKKVEPKSCDKTHT

**Her2 Fab^H^ (pSFV4)**

SDIQMTQSPSSLSASVGDRVTITCRASQDVNTAVAWYQQKPGKAPKLLIYSASFLYSGVPSRFSGSRSGTDFTLTISSLQPEDFATYYCQQHYTTPPTFGQGTKVEIKRTVAAPSVFIFPPSDEQLKSGTASVVCLLNNFYPREAKVQWKVDNALQSGNSQESVTEQDSKDSTYSLSSTLTLSKADYEKHKVYACEVTHQGLSSPVTKSFNRGEC/MKKNIAFLLASMFVFSIATNAYAEISEVQLVESGGGLVQPGGSLRLSCAASGFNIKDTYIHWVRQAPGKGLEWVARIYPTNGYTRYADSVKGRFTISADTSKNTAYLQMNSLRAEDTAVYYCSRWGGDGFYAMDYWGQGTLVTVSSASTKGPSVFPLAPSSKSTSGGTAALGCLVKDYFPEPVTVSWNSGALTSGVHTFPAVLQSSGLYSLSSVVTVPSSSLGTQTYICNVNHKPSNTKVDKKVEPKSCDKTHT

**EGFR Fab^LRT^ (pSFV4)**

SDIQMTQSPSSLSASVGDRVTITCQASQDISNYLNWYQQKPGKAPKLLIYDASNLETGVPSRFSGSGSGTDFTFTISSLQPEDIATYFCQHFDHLPLAFGGGTKVEIKRTVAAPSVFIFPPSDLRTGTASVVCLLNNFYPREAKVQWKVDNALQSGNSQESVTEQDSKDSTYSLSSTLTLSKADYEKHKVYACEVTHQGLSSPVTKSFNRGEC/QVQLQESGPGLVKPSETLSLTCTVSGGSVSSGDYYWTWIRQSPGKGLEWIGHIYYSGNTNYNPSLKSRLTISIDTSKTQFSLKLSSVTAADTAIYYCVRDRVTGAFDIWGQGTMVTVSSAPTKGPSVFPLAPSSKSTSGGTAALGCLVKDYFPEPVTVSWNSVALTSGVHTFPAVLQSSGLYSLSSVVTVPSSSLGTQTYICNVNHKPSNTKVDKKVEPKSCDKTHT

**MJ20 Fab^H^ (pSFV4)**

SDIQMTQSPSSLSASVGDRVTITCRASQSVSSAVAWYQQKPGKAPKLLIYSASSLYSGVPSRFSGSRSGTDFTLTISSLQPEDFATYYCQQSSSSLITFGQGTKVEIKRTVAAPSVFIFPPSDEQLKSGTASVVCLLNNFYPREAKVQWKVDNALQSGNSQESVTEQDSKDSTYSLSSTLTLSKADYEKHKVYACEVTHQGLSSPVTKSFNRGEC/EISEVQLVESGGGLVQPGGSLRLSCAASGFNISYSSIHWVRQAPGKGLEWVASIYSYSGYTSYADSVKGRFTISADTSKNTAYLQMNSLRAEDTAVYYCARSYWYHVGSWHYTGMDYWGQGTLVTVSSASTKGPSVFPLAPSSKSTSGGTAALGCLVKDYFPEPVTVSWNSGALTSGVHTFPAVLQSSGLYSLSSVVTVPSSSLGTQTYICNVNHKPSNTKVDKKVEPKSCDKTHT

**PGA1-hFc (pSCSTa)**

TPAVTTYKLVINGRTLSGYTTTTAVDAATAEKVFKQYAYVHEVDGEWTYDDATKTFTVTEKPEKLGGGGSGGGSGGGGSGGGGSGGGGSSSGSSCPPCPAPELLGGPSVFLFPPKPKDTLMISRTPEVTCVVVDVSHEDPEVKFNWYVDGVEVHNAKTKPREEQYNSTYRVVSVLTVLHQDWLNGKEYKCKVSNKALPAPIEKTISKAKGQPREPQVYTLPPSREEMTKNQVSLTCLVKGFYPSDIAVEWESNGQPENNYKTTPPVLDSDGSFFLYSKLTVDKSRWQQGNVFSCSVMHEALHNHYTQKSLSLSPGK

**PGLM-hFc (pSCSTa)**

TPAVTTYKLVINGRTLSGYTTTTAVDAATAEKVFKQLGMMRSVDGEWTYDDATKTFTVTEKPEKLGGGGSGGGSGGGGSGGGGSGGGGSSSGSSCPPCPAPELLGGPSVFLFPPKPKDTLMISRTPEVTCVVVDVSHEDPEVKFNWYVDGVEVHNAKTKPREEQYNSTYRVVSVLTVLHQDWLNGKEYKCKVSNKALPAPIEKTISKAKGQPREPQVYTLPPSREEMTKNQVSLTCLVKGFYPSDIAVEWESNGQPENNYKTTPPVLDSDGSFFLYSKLTVDKSRWQQGNVFSCSVMHEALHNHYTQKSLSLSPGK

**PGA1-mFc (pSCSTa)**

TPAVTTYKLVINGRTLSGYTTTTAVDAATAEKVFKQYAYVHEVDGEWTYDDATKTFTVTEKPEKLGGGGGGSGGGGSGGGGSCPPCKCPAPNLLGGPSVFIFPPKIKDVLMISLSPIVTCVVVDVSEDDPDVQISWFVNNVEVHTAQTQTHREDYNSTLRVVSALPIQHQDWMSGKEFKCKVNNKDLPAPIERTISKPKGSVRAPQVYVLPPPEEEMTKKQVTLTCMVTDFMPEDIYVEWTNNGKTELNYKNTEPVLDSDGSYFMYSKLRVEKKNWVERNSYSCSVVHEGLHNHHTTKSFSRTPGK

**PGLM-mFc (pSCSTa)**

TPAVTTYKLVINGRTLSGYTTTTAVDAATAEKVFKQLGMMRSVDGEWTYDDATKTFTVTEKPEKLGGGGGGSGGGGSGGGGSCPPCKCPAPNLLGGPSVFIFPPKIKDVLMISLSPIVTCVVVDVSEDDPDVQISWFVNNVEVHTAQTQTHREDYNSTLRVVSALPIQHQDWMSGKEFKCKVNNKDLPAPIERTISKPKGSVRAPQVYVLPPPEEEMTKKQVTLTCMVTDFMPEDIYVEWTNNGKTELNYKNTEPVLDSDGSYFMYSKLRVEKKNWVERNSYSCSVVHEGLHNHHTTKSFSRTPGK
